# FERONIA Confers Resistance to Photooxidative Stress in Arabidopsis

**DOI:** 10.3389/fpls.2021.714938

**Published:** 2021-07-15

**Authors:** Seung Yong Shin, Ji-Sun Park, Hye-Bin Park, Ki-Beom Moon, Hyun-Soon Kim, Jae-Heung Jeon, Hye Sun Cho, Hyo-Jun Lee

**Affiliations:** ^1^Plant Systems Engineering Research Center, Korea Research Institute of Bioscience and Biotechnology, Daejeon, South Korea; ^2^Department of Functional Genomics, KRIBB School of Bioscience, University of Science and Technology, Daejeon, South Korea; ^3^Department of Biosystems and Bioengineering, KRIBB School of Biotechnology, University of Science and Technology, Daejeon, South Korea

**Keywords:** FERONIA, photoprotection, photooxidative damage, stress resistance, ROS

## Abstract

Plants absorb light energy required for photosynthesis, but excess light can damage plant cells. To protect themselves, plants have developed diverse signaling pathways which are activated under high-intensity light. Plant photoprotection mechanisms have been mainly investigated under conditions of extremely high amount of light; thus, it is largely unknown how plants manage photooxidative damage under moderate light intensities. In the present study, we found that FERONIA (FER) is a key protein that confers resistance to photooxidative stress in plants under moderate light intensity. FER-deficient mutants were highly susceptible to increasing light intensity and exhibited photobleaching even under moderately elevated light intensity (ML). Light-induced expression of stress genes was largely diminished by the *fer-4* mutation. In addition, excitation pressure on Photosystem II was significantly increased in *fer-4* mutants under ML. Consistently, reactive oxygen species, particularly singlet oxygen, accumulated in *fer-4* mutants grown under ML. FER protein abundance was found to be elevated after exposure to ML, which is indirectly affected by the ubiquitin-proteasome pathway. Altogether, our findings showed that plants require FER-mediated photoprotection to maintain their photosystems even under moderate light intensity.

## Introduction

Light is an essential energy source during plant photosynthesis, but excess light can cause damage to the photosynthetic machinery (Murchie and Niyogi, 2011; Takahashi and Badger, 2011). Excess excited energy due to high amount of light leads to electron transfer to oxygen, thus generating various types of reactive oxygen species (ROS) including hydrogen peroxide, superoxide, and singlet oxygen (Logan et al., 2006; Takahashi and Badger, 2011; Agati et al., 2013). Plants have both physical and chemical responses to avoid photodamage. Under high light conditions, plant leaves are rearranged and chloroplasts are repositioned to avoid direct exposure to light (Kasahara et al., 2002; Takahashi and Badger, 2011). Plants with mutations in chloroplast avoidance movement are highly susceptible to high light and exhibit photobleaching (Kagawa et al., 2001; Kasahara et al., 2002), suggesting that physical avoidance of light is important for managing photodamage. Meanwhile, plants have developed molecular systems that convert excess excited energy to thermal energy, which is harmless to plant cells (Pascal et al., 2005; Murchie and Niyogi, 2011). In addition, diverse antioxidants, including flavonoids, ascorbate, and glutathione, along with antioxidant enzymes such as superoxide dismutase, ascorbate peroxidase, and glutathione reductase scavenge destructive ROS generated as a consequence of high light intensity (Logan et al., 2006; Agati et al., 2013).

To date, most studies have been performed under high light conditions (500–1,500 μmol m^–2^ s^–1^) to investigate plant photoprotection responses (Kasahara et al., 2002; Hubbart et al., 2018; Garcia-Molina and Leister, 2020). However, it is largely unknown how plants manage photodamage under moderate light conditions (60–150 μmol m^–2^ s^–1^), because plants exhibit relatively normal growth phenotype in these conditions. While plants grown under moderate light conditions (100 μmol m^–2^ s^–1^) show similar photosystem II (PSII) excitation pressure to those grown under low light conditions (30 μmol m^–2^ s^–1^), exposure to high light (500 μmol m^–2^ s^–1^) triggers a large increase in PSII excitation pressure (Mikko et al., 2006). In addition, high light treatment to the Arabidopsis leaves grown under moderate light conditions decreases maximum PSII quantum yield (Mikko et al., 2006). These results indicate that unlike moderate light, high light triggers severe photoinhibition in plants. Application of high light (1000 μmol m^–2^ s^–1^) to the Arabidopsis changes expression of more than 180 genes compared with the moderate light (100 μmol m^–2^ s^–1^; Rossel et al., 2002), suggesting that different signaling pathways are activated by different light intensities.

FERONIA (FER) is a receptor-like kinase localized in the plasma membrane, and it possesses extracellular and cytoplasmic domains (Höfte, 2015; Li et al., 2016a). Pleiotropic phenotypes of *fer* mutants indicated that FER has diverse functions in plant growth and development including hypocotyl and root elongation, root hair development, and flowering time (Deslauriers and Larsen, 2010; Duan et al., 2010; Wang et al., 2020; Zhu et al., 2020). Rapid alkalinization factor 1 (RALF1) is a small peptide known as a growth regulator recognized by FER (Haruta et al., 2014). The binding of RALF1 to the extracellular domain induces FER phosphorylation (Haruta et al., 2014). The RALF1-FER complex is an active kinase which phosphorylates interacting proteins, including H^+^-adenosine triphosphatases (H^+^-ATPases), RNA-binding proteins, and eukaryotic translation initiation factors (Haruta et al., 2014; Li et al., 2016a; Wang et al., 2020; Zhu et al., 2020). RALF1-FER signaling regulates cell growth. Treatment of wild type seedlings with RALF1 peptide inhibits cell expansion and negatively affects hypocotyl and root elongation, but its effects are diminished by *fer* mutations (Bergonci et al., 2014; Haruta et al., 2014; Li et al., 2016a). The RALF1-FER complex phosphorylates H^+^-ATPases and negatively regulates their H^+^-pumping activity, which plays a key role in cell growth regulation (Haruta et al., 2014). The interaction of FER with growth-regulating phytohormones, such as brassinosteroid (BR) and ethylene, further suggests the role of FER in shaping plant architecture (Deslauriers and Larsen, 2010). FER is also important for fertilization, as it induces the production of ROS in the ovules at the entrance of the pollen tube in order for the pollen tube to rupture and thereby facilitate sperm release (Duan et al., 2014). These observations indicate that FER is a multi-functional protein which affects growth and reproduction in different tissues.

Plant responses to environmental signals include the FER signaling pathways. FER enhances plant resistance against bacterial pathogens by phosphorylation and destabilization of MYC2, which is a key transcription factor in jasmonic acid signaling (Guo et al., 2018). In contrast, FER is a target protein of the fungus *Fusarium oxysporum* which uses the RALF peptide to increase the extent of fungal infection (Masachis et al., 2016). In addition to biotic stress responses, FER controls plant stress responses under abiotic conditions, including salt, gravity, and mechanical stress (Shih et al., 2014; Chen et al., 2016; Feng et al., 2018; Dong et al., 2019). For example, the extracellular domain of FER interacts with pectin to control cell wall integrity and induce resistance responses against salt stress through a transient increase in cytosolic Ca^2+^ concentration (Feng et al., 2018). Mechanical stimulation, such as touch, also generates Ca^2+^ signatures in the root cells through FER, which is important for root obstacle avoidance (Shih et al., 2014). FER is involved in general stress responses by interacting with signaling pathways of plant stress hormone abscisic acid (ABA) (Yu et al., 2012), indicating that FER has a key role in plant-environmental interactions.

In the present study, we found that plants need to alleviate photodamage even under moderate light intensity, and that FER is required for these responses. FER mutation caused photobleaching and cell death under moderately elevated light intensity (ML), but did not affect chlorophyll content and survival under dim light (DL). Induction of stress-responsive gene expression and ROS scavenging after exposure to ML were dependent on FER functions. In addition, excitation pressure on PSII was increased in *fer-4* mutants. Meanwhile, the FER protein abundance was elevated by increased light intensity. Our findings indicate which mechanisms plants use to manage and protect themselves from moderate light intensity.

## Materials and Methods

### Plant Materials and Growth Conditions

*Arabidopsis thaliana* ecotype Columbia (Col-0) was used for the assays. The *fer-4* (N69044; Yu et al., 2012), *the1-4* (CS829966; Guo et al., 2009), *llg1-2* (CS66106; Li et al., 2015), *gef1 gef4 gef10* (N69175; Li et al., 2016c), *abi1-2* (CS859686; Chen et al., 2016), *abi2-2* (N515166; Chen et al., 2016), *aba2-1* (N156; Léon-Kloosterziel et al., 1996c), *bri1-201* (N9532; Bouquin et al., 2001), *gapc1-1* (Salk_129091; Yang et al., 2015), *gapc2-2* (Salk_016539; Yang et al., 2015), and *ralf1-2* (N589792; Feng et al., 2018) mutants were obtained from the Nottingham Arabidopsis Stock Centre (NASC, Nottingham, United Kingdom) and the Arabidopsis Biological Resource Center (ABRC, Ohio State University, Columbus, OH, United States). Their seeds were surface-sterilized in 75% (v/v) ethanol with 0.03% (v/v) Triton X-100 and then washed twice using 70% (v/v) ethanol before stratification at 4°C. After 3 days of stratification, the seeds were transferred to a growth room set at 24°C with 40–50% humidity under long-day conditions. The seedlings were grown on 1/2 × Murashige and Skoog-agar (MS-agar) plates containing 0.7% (w/v) plant agar without sucrose supplementation. The plates were exposed to different light intensities ranging from 1.4 to 154 μmol m^–2^ s^–1^ using fluorescent FL40EX-D tubes (Focus, Bucheon, South Korea).

To generate *FER_*pro*_:FER-MYC* transgenic plants, ∼1,240 bp upstream of the *FER* gene and FER-coding sequence fragments, which have been described previously (Shih et al., 2014), were amplified and combined into a modified myc-pBA vector (Kim et al., 2017) using AvrII-BamHI and BamHI-AscI restriction enzyme sites. The vector constructs were transformed into Col-0 plants using the *Agrobacterium*-mediated floral dip method (Zhang et al., 2006). Complementation lines were generated by crossing *FER_*pro*_:FER-MYC* transgenic plants with *fer-4* mutants.

### Chlorophyll Content Measurement

For chlorophyll content measurement, fresh weights of the seedlings were measured, and 20–40 mg of plant materials were incubated in the 1 mL of methanol at 4°C for ∼16 h. Absorbance was measured at 652 nm (A652) and 665 nm (A665) using a spectrophotometer (Beckman Coulter, Brea, CA, United States). Chlorophyll content was calculated as described previously (Porra et al., 1989). Chlorophyll a and b contents were calculated as follows:

$$\begin{array}{c} Chlorophylla=16.29\times A665-8.54\times A652 \end{array}$$

$$\begin{array}{c} Chlorophyllb=30.66\times A652-13.58\times A665 \end{array}$$

The sum of chlorophyll a and b contents was divided by fresh weight of plant materials. Relative values were calculated to compare the relative chlorophyll content in each treatment.

### Trypan Blue Staining

The seedlings grown on MS-agar plates under long-day conditions with ML or DL (154 or 14 μmol m^–2^ s^–1^, respectively) were subjected to trypan blue staining. The seedlings were immersed in trypan blue solution containing 10 mL lactic acid (85% w/w), 10 ml phenol (pH 7.5–8.0), 10 mL glycerol, 10 mL distilled water, and 40 mg trypan blue. The seedlings were incubated in the solution for 1 h at room temperature. After the staining, the solutions were removed and plant materials were washed twice with 98–100% (v/v) ethanol. Then, the seedlings were incubated in ethanol until their tissues became colorless. Plant materials were mounted on slide glasses and photographed using a Nikon D5600 digital camera.

### Reverse-Transcription Quantitative PCR (RT-qPCR)

Total RNA was extracted from the seedlings using the RNeasy Plant Mini Kit (QIAGEN, Hilden, Germany). Reverse transcription was performed using the TOPscript cDNA Synthesis Kit (Enzynomics, Daejeon, South Korea). Reverse-transcription quantitative PCR (RT-qPCR) reactions were performed using a CFX Connect Real-Time PCR Detection System (Bio-Rad, Hercules, CA, United States) in 96-well plates. TOPreal qPCR PreMIX (Enzynomics) was used for qPCR. The primers used in the RT-qPCR reactions are listed in Supplementary Table 2. The reference gene *UBQ10* (AT4G05320) was included for internal control. The comparative ΔΔC_*T*_ method was used to evaluate the relative values of each amplified product in the reaction according to the manufacturer’s instructions. The threshold cycle (C_*T*_) was automatically determined for each reaction by the system. Melting curve analysis was performed to evaluate the specificities of each primer.

### RNA-Sequencing

Total RNA was extracted from the Col-0 and *fer-4* seedlings grown on MS-agar plates under long-day conditions for 7 days at ML. Three biological replicates were analyzed. The RNA samples were subjected to RNA sequencing by LAS. Raw RNA-sequencing data were produced through MGISEQ-2000. Sequencing adapters and low quality bases in the raw reads were trimmed and then high quality reads were mapped to the TAIR10 reference genome^1^. Expression of genes in Col-0 and *fer-4* seedlings was compared and the genes with *Q*-value ≤ 0.01 and fold change ≥ 2 were regarded as differentially expressed genes (DEGs). GO analysis was performed using the Biological Networks Gene Ontology tool (BiNGO) with Benjamini-Hochberg-corrected *P* < 0.05. The significantly overrepresented GO terms were displayed with the network diagram.

### Chlorophyll Fluorescence Measurement

The Col-0 and *fer-4* seedlings were grown on MS-agar plates under long-day conditions with three different light conditions: DL for 7 days, DL for 4 days and ML for 3 days, and ML for 7 days. Average fluorescence levels were measured with FluorCam 800MF (PSI, Drasov, Czechia) with an optimized kinetic quenching protocol. The zero fluorescence level was measured under the low intensity of measuring light (F_*o*_), and then the maximum fluorescence level was measured with a saturating flash of light (F^*o*^_*m*_). Then, actinic light was applied. After a period of time, fluorescence level was measured before (F_*t*_) and with (F’_*m*_) application of another saturating light flash. Then, actinic light was turned off and zero fluorescence level was measured (F’_*o*_). Photochemical quenching parameters were calculated as described previously (Maxwell and Johnson, 2000):

$$\begin{array}{c} MaximumquantumyieldofPSII=\frac{F_{m}^{o}-F_{o}}{F_{m}^{o}} \end{array}$$

$$\begin{array}{c} QuantumyieldofPSII=\frac{F_{m}^{\prime }-F_{t}}{F_{m}^{\prime }} \end{array}$$

$$\begin{array}{c} ProportionofopenPSII\left(qP\right)=\frac{F_{m}^{\prime }-F_{t}}{F_{m}^{\prime }-F_{o}^{\prime }} \end{array}$$

$$\begin{array}{c} ExcitationpressureonPSII=1-qP \end{array}$$

$$\begin{array}{c} NPQ=\frac{F_{m}^{o}-F_{m}^{\prime }}{F_{m}^{\prime }} \end{array}$$

### ROS Staining

Seedlings grown on MS-agar plates under long-day conditions with ML or DL were subjected to ROS staining. For 3,3′-diaminobenzidine (DAB) staining, the seedlings were immersed in DAB staining solution containing 40 mg DAB and 20 μL Tween 20 in 40 mL distilled water, and the solution with the seedlings was subjected to gentle vacuuming for 5 min. The seedlings were then incubated for ∼8 h in the dark at room temperature, after which the DAB staining solution was removed. After washing twice with 70% (v/v) ethanol, plant materials were incubated for 24 h until their tissues became colorless. The plant materials were mounted on slide glasses and photographed using a Nikon D5600 digital camera. The ImageJ software^2^ was used to quantify the staining intensity. For nitroblue tetrazolium (NBT) staining, the seedlings were immersed in NBT staining solution containing 70 mg NBT and 13 mg sodium azide in 20 mL of 10 mM potassium phosphate buffer (pH 7.8). The plant materials were subjected to vacuuming for 2 min after which they were incubated for 2 h in the dark at room temperature. The NBT staining solution was then removed and the plant materials were washed twice with 70% (v/v) ethanol. The plant materials were mounted on slide glasses and photographed using a Nikon D5600 digital camera. The ImageJ software was used to quantify the staining intensity.

For singlet oxygen sensor green (SOSG) staining, the seedlings were immersed in SOSG staining solution containing 100 μM SOSG in 10 mM potassium phosphate buffer (pH7.8). After 10 min of incubation, the plant materials were mounted on slide glasses and SOSG signals were observed using an LSM 800 confocal microscope (Carl Zeiss, Jena, Germany). The excitation and emission wavelengths were 488 and 500–600 nm, respectively. For chlorophyll autofluorescence measurement, excitation and emission wavelengths were 561 and 560–700 nm, respectively. Fluorescence images were analyzed using the ZEN 2.5 LITE software. The ImageJ software was used to quantify the staining intensity.

### Analysis of Ca^2+^ Signals

Ca^2+^ signals were visualized using the Fluo-3 AM calcium indicator (Thermo Fisher Scientific, Waltham, MA, United States) as described previously (Wang et al., 2016). The seedlings grown on MS-agar plates under long-day conditions with ML or DL were transferred to a Fluo-3 staining solution containing 10 μM Fluo-3 AM in 10 mM Tris-MES (pH 6.1). Plant materials were then incubated for 2 h in the dark. After staining, the plant materials were washed twice with 10 mM Tris-MES (pH 6.1) and mounted on slide glasses. The plant materials were incubated for another hour in the dark for resting touch-induced Ca^2+^ signals. An LSM 800 confocal microscope (Carl Zeiss) was used to analyze Fluo-3 AM signals with excitation and emission wavelengths of 506 and 500–550 nm, respectively. For chlorophyll autofluorescence measurement, excitation and emission wavelengths were 561 and 560–700 nm, respectively. Fluorescence images were analyzed using the ZEN 2.5 LITE software.

### Analysis of Chloroplast Avoidance

The seedlings grown on MS-agar plates under long-day conditions with dim light for 6 days were incubated in the dark for 16 h and then exposed to DL or ML for 3 h. The adaxial side of the cotyledons was observed using an LSM 800 confocal microscope (Carl Zeiss) to analyze chloroplast avoidance. Excitation and emission wavelengths were 561 and 560–700 nm, respectively. Fluorescence images were analyzed using the ZEN 2.5 LITE software.

### Immunoblot Assays

The aerial parts of the *FER_*pro*_:FER-MYC* transgenic seedlings grown on MS-agar plates under long-day conditions with ML or DL were harvested and ground in liquid nitrogen and 1 volume of 2 × SDS-loading buffer containing 150 mM Tris–HCl (pH 6.8), 4.8% (w/v) SDS, 24% (v/v) glycerol, and 672 mM β-mercaptoethanol was added for total protein extraction. The obtained protein extracts were used for SDS-polyacrylamide gel electrophoresis (SDS-PAGE) using protein electrophoresis equipment (Bio-Rad). The proteins were then transferred to Immobilon-P polyvinylidene difluoride membranes (Millipore, Burlington, MA, United States). Anti-MYC (Millipore, Cat. No. 05-724) and anti-α-tubulin (Sigma-Aldrich, St. Louis, MO, United States, Cat. No. T5168) were used to detect FER-MYC fusion and tubulin proteins, respectively.

### Analysis of Protein Ubiquitination

Col-0 and *FER_*pro*_:FER-MYC* seedlings were ground in liquid nitrogen and 2 volumes of IP buffer containing 50 mM Tris–HCl (pH 7.5), 150 mM NaCl, 10% (v/v) glycerol, 5 mM EDTA, 1% (v/v) Triton X-100, 1% (v/v) NP-40, 1 × protease inhibitor cocktail (Sigma-Aldrich), and 50 μM MG132 (Sigma-Aldrich) were added for total protein extraction. The 5% (v/v) of protein extracts were transferred to new tubes as input controls. They were then mixed with 40 μL of protein G magnetic beads (Bio-Rad) and incubated for 1 h at 4°C with rotation to remove non-specific binding proteins. During the incubation, 5 μg of anti-MYC antibody was mixed with 40 μL of protein G magnetic beads for 1 h at 4°C with rotation to build antibody-protein G magnetic bead complex. After removing the free beads from protein extracts, antibody-bead complexes were added to protein extracts and incubated for 2 h at 4°C with rotation for immunoprecipitation. After incubation, the IP buffer was removed and the beads were washed two times with 1 mL of low salt wash buffer containing 50 mM Tris–HCl (pH 7.5), 150 mM NaCl, 10% (v/v) glycerol, 5 mM EDTA, 1% (v/v) Triton X-100 and 1% (v/v) NP-40. The beads were then washed twice with 1 mL of high salt wash buffer containing 50 mM Tris–HCl (pH 7.5), 500 mM NaCl, 10% (v/v) glycerol, 5 mM EDTA, 1% (v/v) Triton X-100 and 1% (v/v) NP-40. After washing, the wash buffers were removed and the immunoprecipitated proteins were eluted with 100 μL of 2 × SDS loading buffer. The proteins were immunoblotted to analyze the polyubiquitination of FER-MYC fusion proteins using anti-MYC and anti-ubiquitin antibodies (Santa Cruz Biotechnology, Dallas, TX, United States, Cat. No. sc-8017).

### Polysome Profiling Assay

The Col-0 seedlings grown on MS-agar plates under long-day conditions for 7 days at DL or ML were used for polysome profiling. Polysomes were fractionated over sucrose gradients as described previously (Lecampion et al., 2016). Sucrose gradients (20–50%) were prepared in 13.5 ml ultracentrifuge tube. For cytosolic extracts, ∼100 mg of Col-0 seedlings were ground in liquid nitrogen and then 1.2 ml of pre-cooled polysome buffer containing 160 mM Tris–HCl pH 8.4, 80 mM KCl, 40 mM MgCl_2_, 5.26 mM EGTA, 0.5% (v/v) Octylphenoxy poly(ethyleneoxy)ethanol, branched, 50 μg ml^–1^ cycloheximide, and 50 μg ml^–1^ chloramphenicol was added. The mixture was centrifuged at 16,000 × *g* for 15 min at 4°C and the supernatant was transferred to a new tube. Then, 100 μl of the supernatant was collected as input and 1 ml of the supernatant was loaded on top of the sucrose gradient. The mixture was centrifuged at 175,000 × *g* for 2 h 45 min at 4°C using ultracentrifuge. Then, the centrifuged mixture was subjected to the gradient collection system with continuous measurement of absorbance at 280 nm. Among 11 fractions, we collected bottom 8–11 fractions for extraction of polysome-associated mRNAs. RNA was extracted from the input and polysome fractions using TRIzol (Thermo Fisher Scientific). Translation efficiency was analyzed as described previously (Zhu et al., 2020). The relative content of the *FER* transcripts in input and polysome fractions was measured by RT-qPCR. Then, the relative proportion of the polysome-associated *FER* transcripts in the total *FER* transcripts was calculated. We used *eIF4A* as the reference gene.

### Statistical Analysis

All statistical methods as well as the number of biological replicates in each assay are annotated in the figure legends. To determine statistically significant differences, one-way analysis of variance (ANOVA) with *post hoc* Tukey’s test and Student’s *t*-test were performed using Rstudio and Excel software, respectively.

## Results

### FER Is Required for Survival Under ML

**FIGURE 1:**
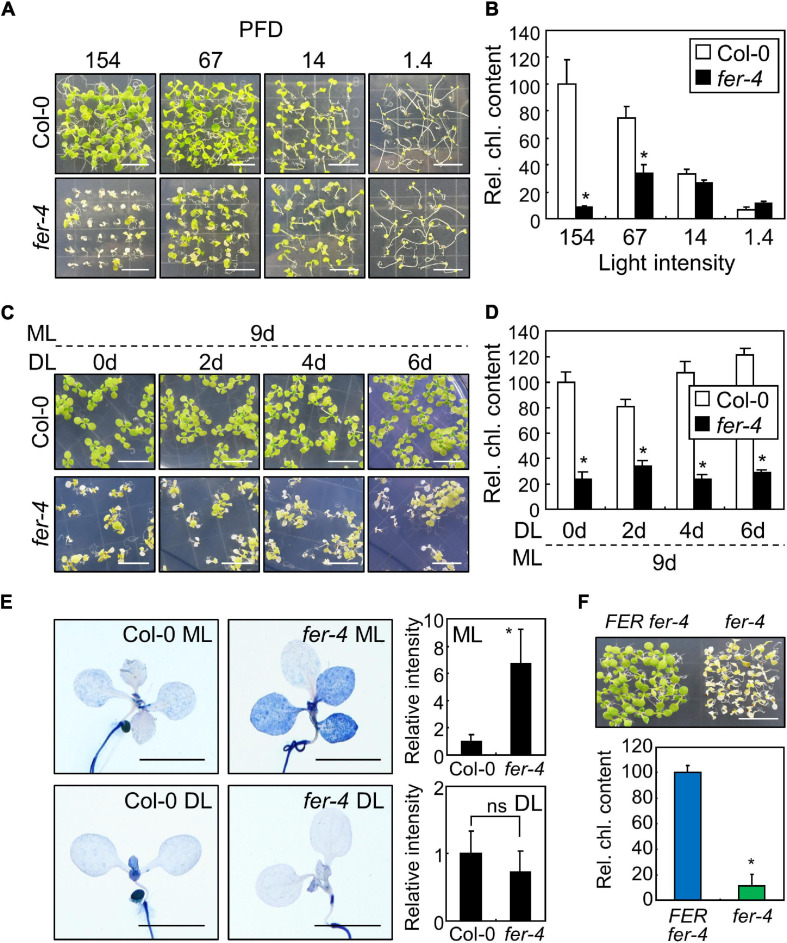
Photobleaching of the *fer* mutants under ML. In bar graphs, whiskers indicate standard deviations (SD). **(A,B)** Phenotypes of the *fer-4* mutants under different light conditions. Seedlings were grown for 10 days under different light intensities **(A)**. *X*-axis numbers indicate photon flux density (PFD) (μmol m^–2^ s^–1^). Whole seedlings were harvested for chlorophyll measurement **(B)**. Relative chlorophyll content (Rel. chl. content) was measured. Biological triplicates were averaged and statistically analyzed using Student’s *t*-test (*P < 0.01; difference from Col-0). Scale bars indicate 1 cm. **(C,D)** Effects of ML at different seedling ages. Seedlings were grown for the indicated periods under DL (14 μmol m^–2^ s^–1^). Seedlings were then transferred to ML (154 μmol m^–2^ s^–1^) and incubated for 9 days **(C)**. Whole seedlings were harvested for chlorophyll measurement **(D)**. Biological triplicates were averaged and statistically analyzed using Student’s *t*-test (*P < 0.01; difference from Col-0). Scale bars indicate 1 cm. **(E)** Trypan blue staining. Ten-day-old seedlings grown under ML or DL were subjected to Trypan blue staining. Trypan blue signal intensities from cotyledons and leaves were measured using ImageJ software. For DL, only those from the cotyledons were measured. Ten replicates were averaged and statistically analyzed using Student’s *t*-test (*P < 0.01; difference from Col-0). ns, not significant. Scale bars indicate 0.2 cm. **(F)** Complementation of *fer-4* with the *FER* gene. *FER_*pro*_:FER-MYC* × *fer-4* (*FER fer-4*) and *fer-4* seedlings were grown under ML for 10 days. Whole seedlings were harvested for chlorophyll measurements. Biological triplicates were averaged and statistically analyzed using Student’s *t*-test (*P < 0.01; difference from *FER fer-4*). Scale bar indicates 1 cm.

In our previous research, we used FER-deficient *fer-4* mutants to examine root obstacle avoidance (Lee et al., 2020). During the experiments, we found that the leaf color of *fer-4* seedlings faded and growth decreased when the seedlings were grown for more than 10 days under the growth conditions with the light intensity of 67 μmol m^–2^ s^–1^ (Figure 1A). Chlorophyll loss is usually observed in wild type plants exposed to high light intensity (Kasahara et al., 2002; Havaux et al., 2005). However, it is unknown whether FER is related to photobleaching under moderate light conditions. Therefore, we investigated the phenotype of *fer-4* seedlings under different light conditions. To examine the effects of light intensity, we exposed the *fer-4* seedlings to different light intensities for 10 days. Phenotypes of Col-0 and *fer-4* seedlings were similar under DL (14 μmol m^–2^ s^–1^), but *fer-4* seedlings exhibited photobleaching and retarded growth under ML (154 μmol m^–2^ s^–1^; Figure 1A and Supplementary Figure 1). Consistently, the chlorophyll content in *fer-4* seedlings was significantly reduced by exposure to light of more than 67 μmol m^–2^ s^–1^ (Figure 1B). Because the photobleaching phenotype was clearer at the light intensity of 154 μmol m^–2^ s^–1^ than at 67 μmol m^–2^ s^–1^, we set 154 μmol m^–2^ s^–1^ as ML for following experiments. Next, we performed time-course measurements of chlorophyll content in Col-0 and *fer-4* seedlings grown for 4, 6, and 10 days under ML, and we found that photobleaching could be observed after 10 days of ML treatment (Supplementary Figure 2). Photobleaching was also observed in *fer-4* mutants under short-day conditions (8 h light, 16 h dark) with ML, showing that FER function in light resistance is not dependent on photoperiod (Supplementary Figure 3). These results suggest that FER is required to maintain chlorophyll content, which is important for photosynthesis (Kato et al., 2020).

Chlorophyll biosynthesis occurs within 48 h of germination under long-day conditions (16 h light, 8 h dark), and it is an important step for heterotroph-to-autotroph transition in plants (Mansfield and Briarty, 1996; Ha et al., 2017). During this stage, plants need to induce molecular signals to maintain optimal chlorophyll biosynthesis despite environmental changes (Ha et al., 2017). To investigate whether FER function in photoprotection is dependent on seedling developmental stages, we carried out an ML treatment for 9 days on Col-0 and *fer-4* seedlings that were grown for 0–6 days under DL conditions. Their growth phenotypes and chlorophyll content showed that exposure to ML caused photobleaching in *fer-4* seedlings regardless of seedling age (Figures 1C,D). Next, we analyzed growth phenotype of Col-0 and *fer-4* plants which were grown firstly under DL for 3 weeks and then transferred to ML or left under DL for additional 3 weeks to examine the role of FER at adult stage. While photobleaching phenotype was not observed in *fer-4* plants under ML, growth parameters were significantly decreased in *fer-4* mutants in compared to those in Col-0 plants (Supplementary Figure 4). The adult *fer-4* plants grown under DL also showed reduced growth, but the difference of growth parameters between Col-0 and *fer-4* under DL was much smaller than that under ML (Supplementary Figure 4D). These results suggest that FER is required for photoprotection and maintaining growth performance at both seedling and adult stages.

Photobleaching can result in cell death, because the ROS produced in chloroplasts as a consequence of excess light induce photooxidative damage which not only disrupts photosystems but also affects cell viability (Hideg et al., 1998; Laloi and Havaux, 2015). We examined the role of FER in cell viability under different light intensities using trypan blue staining. Histochemical observations revealed that cell death was observed in both cotyledons and leaves of *fer-4* seedlings grown under ML (Figure 1E). In contrast, the intensity of trypan blue staining was largely reduced in *fer-4* seedlings grown under DL, suggesting that FER-deficient mutants cannot manage photodamage under ML. As we only used a single mutant line, we generated a complementation line by expressing the *FER* gene in the *fer-4* mutant under the control of the native *FER* promoter (*FER_*pro*_:FER-MYC* × *fer-4*). Expression of the *FER* gene in *fer-4* seedlings restored the photobleaching phenotype under ML (Figure 1F), verifying the role of FER in plant photoprotection. Because *fer-4* mutants exhibited a photodamaged phenotype at ML that was far below the usual high light intensity (500–1,500 μmol m^–2^ s^–1^), these results indicate that plants need to manage photodamage even under ML, and that FER plays a key role in these responses.

### FER Induces the Expression of Stress Genes Under ML After Night-Day Transition

**FIGURE 2:**
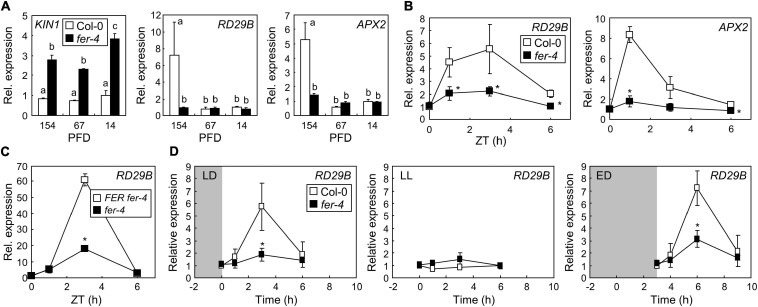
FERONIA (FER) induces the expression of stress genes under ML. Whiskers indicate SD. **(A)** Expression of stress genes in *fer-4* mutants under different light intensities. Seedlings grown under different light intensities for 7 days were harvested at zeitgeber time (ZT) 3. *X*-axis numbers indicate PFD (μmol m^–2^ s^–1^). Biological triplicates were averaged. Letters indicate groups that are statistically significantly different from each other (*P* < 0.05, Tukey’s test). **(B)** Time-course expression of stress genes in *fer-4* mutants. Seedlings were grown under ML for 7 days. Whole seedlings were harvested at ZT0, 1, 3, and 6. Biological triplicates were averaged and statistically analyzed using Student’s *t*-test (*P < 0.05; difference from Col-0). **(C)** Recovery of *RD29B* expression by complementation of *fer-4* with the *FER* gene. *FER fer-4* and *fer-4* seedlings were treated as described in panel **(B)**. Biological triplicates were averaged and statistically analyzed using Student’s *t*-test (*P < 0.05; difference from *FER fer-4*). **(D)** Diurnal expression of the *RD29B* gene. Seedlings grown under long-day conditions at ML for 7 days were incubated in the dark after the end of the light period for 8 h (LD; left panel) or 11 h (ED; right panel). For LL, seedlings grown under long-day conditions at ML for 7 days were kept in light without a dark period (middle panel). Seedlings treated with LD and LL were harvested at the same time points. Seedlings treated with ED were harvested at 0, 1, 3, and 6 h after the end of the dark treatment. Biological triplicates were averaged and statistically analyzed using Student’s *t*-test (*P < 0.05; difference from Col-0).

FERONIA is involved in diverse plant signaling pathways, including ABA signaling, which is necessary for plant responses to high amounts of light (Galvez-Valdivieso et al., 2009). FER is known as a suppressor of ABA signaling, which occurs by activation of ABA INSENSITIVE 2 (ABI2) phosphatase (Yu et al., 2012). In the present study, to investigate whether FER function in ABA signaling is related to photoprotection, we analyzed the expression of ABA-responsive genes encoding KINASE 1 (KIN1) and RESPONSIVE TO DESICCATION 29B (RD29B). In a previous study, the expression of both of these genes in a *fer* mutant was constitutively elevated even under normal conditions and was highly responsive to ABA treatment (Chen et al., 2016). Consistent with this previous study, we found that the expression of *KIN1* was higher in *fer-4* mutants than in Col-0 seedlings under all analyzed light intensity conditions (Figure 2A). However, *KIN1* was not induced by increasing light intensity, suggesting that ABI2-FER signaling module is not involved in plant responses to ML. On the other hand, the expression of *RD29B* in Col-0 seedlings significantly increased under 154 μmol m^–2^ s^–1^ in comparison to that under 67 μmol m^–2^ s^–1^ and 14 μmol m^–2^ s^–1^, but it was not responsive to increasing light intensity in *fer-4* mutants (Figure 2A). Because *RD29B* is a representative gene whose expression is responsive to abiotic stresses, including drought and salt (Chen et al., 2010; Msanne et al., 2011), we analyzed other stress- and ABA-responsive genes in *fer-4* mutants under different light intensities. While *ABI* genes did not show noticeable differences between Col-0 and *fer-4* seedlings (Supplementary Figure 5), *ASCORBATE PEROXIDASE 2* (*APX2*) exhibited similar expression patterns to those of *RD29B*; it was highly induced under ML in Col-0 seedlings but not in *fer-4* mutants (Figure 2A). Increased expression of stress genes, including *RD29B* and *APX2*, is generally related to enhanced stress tolerance (Galvez-Valdivieso et al., 2009; Jin et al., 2013; Shi et al., 2015). These results suggest that plants need to protect themselves even under moderate light conditions possibly by inducing expression of stress genes via FER functions.

As the plants in the present study were grown under long-day conditions, light stress occurred during light periods. To examine how quickly the plants respond to light stress, we analyzed the time-course expression of *RD29B* and *APX2* genes in seedlings grown under ML. We found that both genes significantly increased within 3 h after the end of the dark period, but decreased during the light period until zeitgeber time (ZT) 6 (Figure 2B). However, gene expression was largely suppressed in *fer-4* mutants during the analyzed time periods. Complementation of *fer-4* mutants restored gene expression, confirming the role of FER in stress gene expression under ML (Figure 2C). Because the expression of stress genes transiently increased during early light periods, we hypothesized that the abrupt transition from dark to light might trigger light stress; thus, inducing stress responses in plants. To verify our hypothesis, the seedlings grown for 7 days under long-day and ML conditions were incubated in the dark for 8 h (LD), in continuous light (LL), and in the dark for 11 h (ED) after the end of the light periods. They were then exposed to ML for up to 6 h (Figure 2D). We found that the expression of *RD29B* in Col-0 was elevated 3 h after the end of the dark periods, but in *fer-4* mutants, it was significantly suppressed in both LD and ED (Figure 2D). Notably, *RD29B* expression was not induced in any seedlings, not even in Col-0 seedlings in LL, at all analyzed time points. These results suggest that night-day transition with ML induced stress responses in plants, which was mediated by FER.

**FIGURE 3:**
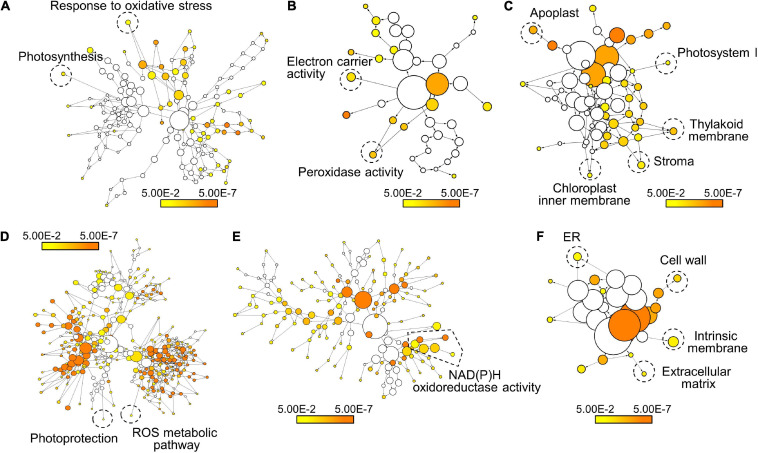
Transcriptome analysis of FER-regulated genes under ML. The Col-0 and *fer-4* seedlings grown for 7 days under ML were harvested for transcriptome analysis. GO analysis of the down-regulated genes **(A–C)** and the up-regulated genes **(D–F)** in *fer-4* mutants was performed using BiNGO. Biological process **(A,D)**, molecular function **(B,E)**, and cellular component **(C,F)** annotations in each set of genes were displayed. Colored nodes indicate GO terms that are significantly overrepresented (Benjamini-Hochberg-corrected *P* < 0.05). Scale bars indicate *P*-values.

To further examine FER-mediated gene expressions under ML, we performed transcriptome analysis in Col-0 and *fer-4* seedlings grown under ML. DEGs were analyzed by comparing RNA-sequencing results of Col-0 and *fer-4* seedlings and then the DEGs were divided by two groups, which are up-regulated and down-regulated genes in *fer-4* mutants (Supplementary Table 1). Next, we analyzed gene ontology (GO) in each group to investigate the function of FER in photoprotection. Because *fer-4* mutants exhibit pleiotropic phenotypes (Deslauriers and Larsen, 2010; Duan et al., 2010; Wang et al., 2020; Zhu et al., 2020), the *fer-4* mutation resulted in altered gene expressions in diverse GO terms. Genes involved in oxidative stress responses and photosynthesis was down-regulated by *fer-4* mutation under ML (Figure 3A and Supplementary Figure 6). Also, genes related to electron carrier and peroxidase activity were down-regulated in the mutants (Figure 3B and Supplementary Figure 7). In addition, genes encoding proteins that are potentially located in Photosystem I, thylakoid membrane, stroma, and chloroplast inner membrane were found to be suppressed by *fer-4* mutation (Figure 3C and Supplementary Figure 8). These results suggest that management of electron transport during the photosynthesis might be disrupted in *fer-4* mutants under ML. GO analysis using up-regulated genes showed that genes involved in photoprotection, ROS metabolic pathway, and NAD(P)H oxidoreductase activity were regulated by FER under ML (Figures 3D,E and Supplementary Figures 9, 10). Notably, GO terms related to the chloroplast compartments were not significantly overrepresented in this group (Figure 3F and Supplementary Figure 11). Overall, transcriptome analysis showed that genes located in chloroplast and related to ROS metabolism and photosynthesis were regulated by FER under ML, suggesting that FER plays a role in managing photosynthetic electron transport for photoprotection.

### FER Is Necessary for Reducing ROS Accumulation Under ML

**FIGURE 4:**
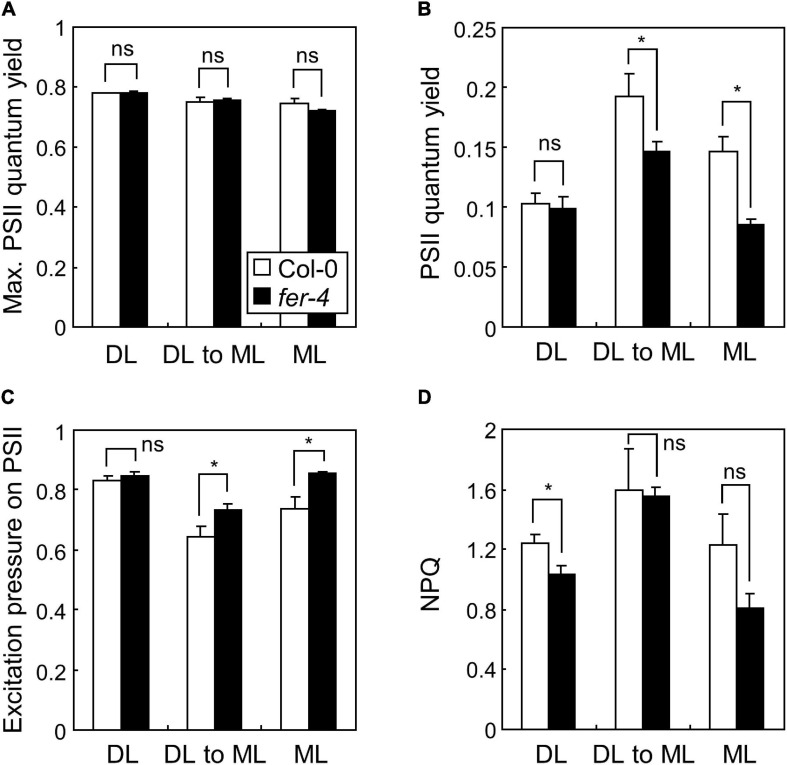
Photosynthetic performance in *fer-4* mutants under different light intensities. Whiskers indicate SD. Seedlings were grown under DL or ML for 7 days, or grown under DL for 4 days and then transferred to ML for three additional days (DL to ML). Maximum PSII quantum yield **(A)**, PSII quantum yield **(B)**, excitation pressure on PSII **(C)**, and NPQ **(D)**, were determined by imaging chlorophyll fluorescence. Three biological replicates were averaged and statistically analyzed using Student’s *t*-test (*P < 0.05; difference from Col-0).

Based on the transcriptome analysis, we performed chlorophyll fluorescence analysis in Col-0 and *fer-4* seedlings under ML to verify whether FER is involved in the photosynthetic electron transport. Because *fer-4* seedlings grown for 7 days under ML were severely damaged, we included seedlings grown under mild conditions: 4 days under DL and then 3 days under ML. Photosynthetic parameters including maximum PSII quantum yield, PSII quantum yield, excitation pressure on PSII, and non-photochemical quenching (NPQ) were analyzed. Maximum PSII quantum yield represents maximum efficiency of PSII when all PSII reaction centers are open (Maxwell and Johnson, 2000). PSII quantum yield represents the proportion of absorbed light energy used for photochemistry in PSII. Excitation pressure on PSII indicates the proportion of the PSII reaction centers that are closed, thus reflects redox state of the PSII electron transport chain (Khanal et al., 2017). NPQ is a parameter related to heat dissipation of absorbed light energy. In our experiments, while maximum PSII quantum yield was not changed by *fer-4* mutation in all conditions (Figure 4A), PSII quantum yield was significantly decreased in *fer-4* mutants when they were exposed to ML (Figure 4B). Notably, excitation pressure on PSII was increased in *fer-4* mutants but NPQ was not altered under ML (Figures 4C,D), showing imbalance in the redox state of the photosynthetic electron transport chain.

**FIGURE 5:**
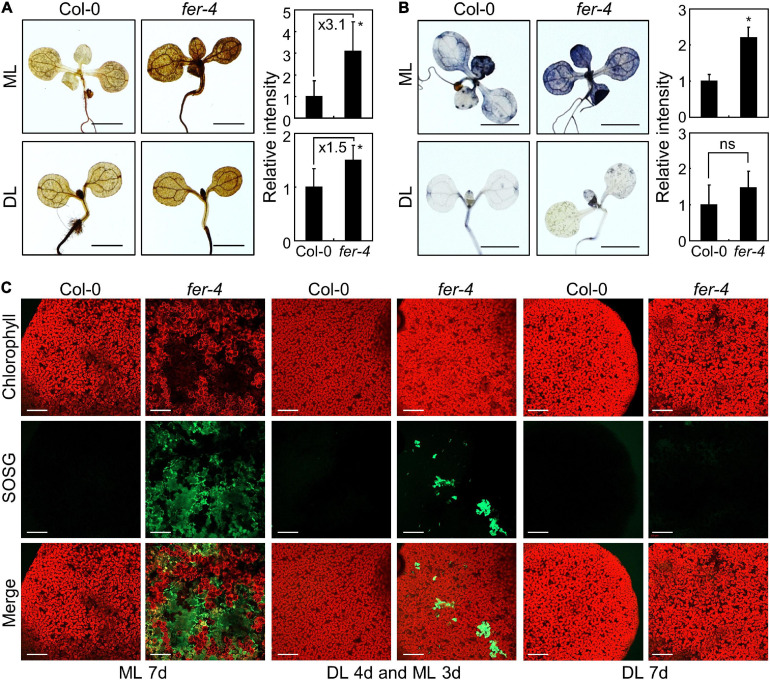
Accumulation of ROS in the *fer-4* mutants under ML. Whiskers indicate SD. **(A)** DAB staining assay. Seedlings grown under ML or DL conditions were subjected to DAB staining at ZT3. Ten replicates were averaged and statistically analyzed using Student’s *t*-test (*P < 0.05; difference from Col-0). Scale bars indicate 0.2 cm. **(B)** NBT staining assay. Seedlings grown at ML or DL were subjected to NBT staining at ZT3. Ten replicates were averaged and statistically analyzed using Student’s *t*-test (*P < 0.05; difference from Col-0). Scale bars indicate 0.2 cm. **(C)** SOSG staining assay. Seedlings were grown under ML or DL for 7 days, or grown under DL for 4 days and then transferred to ML for three additional days before SOSG staining. Seedlings were immersed in SOSG staining solution at ZT3. A confocal microscope was used for detection of SOSG fluorescence. Scale bars indicate 0.2 mm.

Excess excitation pressure could result in generation of ROS (Khanal et al., 2017). Also, photooxidative damage by ROS causes photobleaching and chlorophyll loss in plants (Rossel et al., 2007; Ksas et al., 2015), which were observed in *fer-4* mutants grown under ML (Figures 1A,B and Supplementary Figure 1). We thus analyzed the accumulation of ROS in Col-0 and *fer-4* seedlings grown under ML or DL. We performed histochemical assays using DAB and NBT to detect hydrogen peroxide and superoxide, respectively (Dunand et al., 2007; Daudi and O’Brien, 2012). DAB staining showed that hydrogen peroxide levels were higher in *fer-4* mutants than in Col-0 seedlings under both ML and DL, while the difference was enhanced under ML conditions (Figure 5A). Meanwhile, the results of NBT staining showed that superoxide levels were similar in Col-0 and *fer-4* seedlings under DL, but superoxide accumulated significantly more in the leaves of *fer-4* mutants than in the leaves of Col-0 seedlings under ML (Figure 5B).

In addition to hydrogen peroxide and superoxide, singlet oxygen is the main molecule involved in photooxidative damage and impairment of photosystems in chloroplasts (Hideg et al., 1994, 1998; Woodson, 2019). We used SOSG to detect singlet oxygen with fluorescence (Flors et al., 2006). Under DL, the SOSG signal intensity was low and similar in the leaves of Col-0 and *fer-4* seedlings (Figure 5C). However, SOSG signals in *fer-4* mutants under ML were significantly increased than those in Col-0 seedlings (Figure 5C and Supplementary Figure 12), suggesting that singlet oxygen highly accumulates in *fer-4* mutants under ML. Notably, the regions with high SOSG signals showed low chlorophyll autofluorescence in *fer-4* mutants grown for 7 days under ML (Figure 5C), which is possibly due to chloroplast disruption by singlet oxygen. Altogether, these results indicate that FER protects chloroplasts from accumulation of ROS under moderate light conditions, particularly singlet oxygen.

### Investigation of FER Molecular Functions in Photoprotection

**FIGURE 6:**
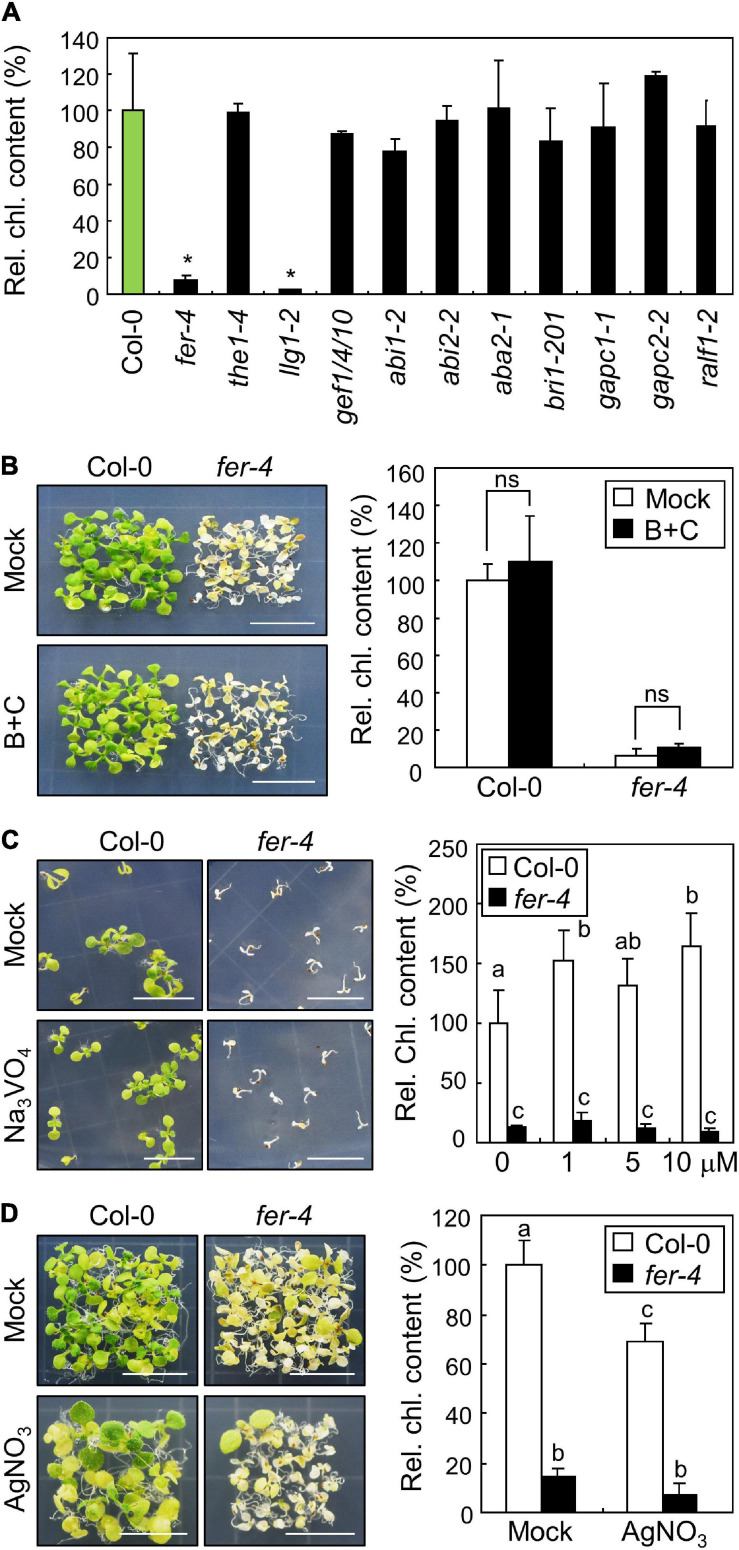
Investigation of FER signaling pathways in photoprotection. Whiskers indicate SD. **(A)** Chlorophyll content of mutants related to FER functions. Seedlings were grown under ML for 10 days. Whole seedlings were harvested for the measurement of chlorophyll content. Biological triplicates were averaged and statistically analyzed using Student’s *t*-test (*P < 0.01; difference from Col-0). **(B)** Effects of borate and calcium supplementation on the *fer-4* mutants. Seedlings were grown on MS-agar plates containing 3 mM H_3_BO_3_ and 5 mM CaCl_2_ (B+C) at ML for 10 days. Whole seedlings were harvested for the measurement of chlorophyll content. Biological triplicates were averaged and statistically analyzed using Student’s *t*-test (*P < 0.01; difference from mock). **(C)** Effects of Na_3_VO_4_ supplementation on the *fer-4* mutants. Seedlings were grown on MS-agar plates containing different concentrations of Na_3_VO_4_ under ML for 10 days. Seedlings treated with mock and 10 μM Na_3_VO_4_ were photographed (left panel). Biological triplicates were averaged. Letters indicate groups that are statistically significantly different from each other (*P* < 0.05, Tukey’s test). **(D)** Effects of AgNO_3_ supplementation on the *fer-4* mutants. Seedlings were grown on MS-agar plates containing 5 μM AgNO_3_ under ML for 10 days. Whole seedlings were harvested for the measurement of chlorophyll content. Biological triplicates were averaged. Letters indicate groups that are statistically significantly different from each other (*P* < 0.05, Tukey’s test).

While we found that FER affects the expression of stress genes and accumulation of ROS under ML, the molecular function of FER in photoprotection is still unknown. FER plays multiple roles in plant development, growth, fertility, and cell wall integrity (Duan et al., 2014; Haruta et al., 2014; Feng et al., 2018; Wang et al., 2020; Zhu et al., 2020). In order to find the connections between known FER functions and photoprotection, we screened the chlorophyll content of mutants related to FER under ML conditions. Because FER is known to be related with THESEUS1 (Guo et al., 2009), LORMLEI-LIKE-GPI-ANCHORED PROTEIN 1 (LLG1; Li et al., 2015), GUANINE NUCLEOTIDE EXCHANGE FACTORs (Yu et al., 2012; Huang et al., 2013), ABA signaling (Yu et al., 2012), BR responses (Deslauriers and Larsen, 2010), GLYCERALDEHYDE-3-PHOSPHATE DEHYDROGENASEs (Yang et al., 2015), and RALF signaling peptides (Haruta et al., 2014), we analyzed the chlorophyll content in *the1-4*, *llg1-2*, *gef1 gef4 gef10*, *abi1-2*, *abi2-2*, *aba2-1*, *bri1-201*, *gapc1-1*, *gapc2-2*, and *ralf1-2* mutants under ML. None of the analyzed mutants showed any defects in chlorophyll content under ML, except for LLG1-defective *llg1-2* mutants (Figure 6A). Chlorophyll content was significantly reduced in *llg1-2* mutants under ML, which was highly similar to the *fer-4* mutants. LLG1 interacts with FER to deliver it to the plasma membrane (Li et al., 2015). In *llg1* mutants, FER fails to move from the endoplasmic reticulum (ER) to the plasma membrane (Li et al., 2015). Therefore, these results suggest that FER functions in photoprotection are related to distinct signaling pathways other than previously known FER-related signaling pathways.

Further, we tried to investigate how the plasma membrane-localized FER alleviates photooxidative stress occurring in chloroplasts. Because treatment with borate and calcium was found to restore the defective growth of *fer-4* roots under salt stress conditions by enhancing cell wall integrity (Feng et al., 2018), we examined the phenotype and chlorophyll content in *fer-4* mutants supplemented with both borate and calcium. However, photobleaching was not restored in *fer-4* mutants after the pharmacological treatment (Figure 6B), suggesting that cell wall integrity defects are not involved in photoprotective role of FER. FER also controls cell expansion by inhibiting proton transport through H^+^-ATPase phosphorylation (Haruta et al., 2014). However, the treatment with the H^+^-ATPase inhibitor sodium orthovanadate did not affect the photobleaching phenotype of *fer-4* mutants under ML (Figure 6C). We also treated Col-0 and *fer-4* seedlings with silver nitrate, because a previous study showed that reduced elongation of hypocotyls in *fer* mutants was restored by silver nitrate, which is an ethylene perception inhibitor (Deslauriers and Larsen, 2010). We found that silver nitrate did not recover the defective phenotype in *fer-4* mutants under ML (Figure 6D). These results suggest that the FER possibly has distinct molecular functions in photoprotection.

**FIGURE 7:**
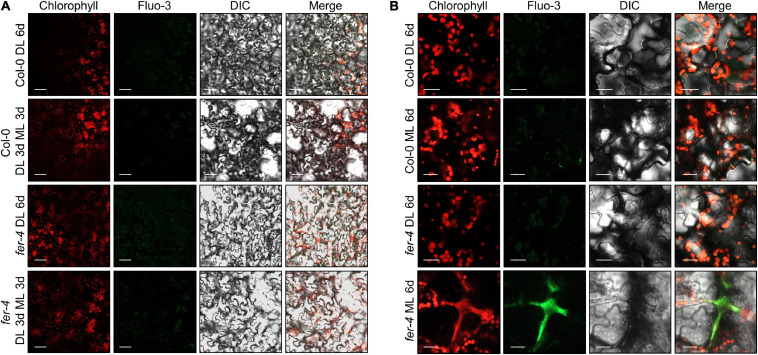
Ca^2+^ signals in the *fer-4* mutants under different light intensities. Seedlings were grown under DL for 3 days and then transferred to ML or left under DL for three additional days **(A)**. To increase ML exposure, seedlings were grown under DL or ML for 6 days **(B)**. After the light treatment, seedlings were subjected to Fluo-3 AM staining. Abaxial side of the leaves was used for observing Ca^2+^ signals. Confocal microscope was used for analyzing fluorescence signals. DIC, differential interference contrast. Size markers indicate 40 μm in panel **(A)** and 20 μm in panel **(B)**.

Cytoplasmic Ca^2+^ influx is an initial step by which plants react to environmental signals, including abiotic and biotic cues (Lecourieux et al., 2006; Kudla et al., 2018). FER controls cytoplasmic Ca^2+^ concentrations in response to salt and touch stimulation (Shih et al., 2014; Feng et al., 2018). We thus examined Ca^2+^ signals using the Ca^2+^ probe Fluo-3 (Wang et al., 2016). Unlike other environmental signals, we could not detect any strong Ca^2+^ signals in Col-0 and *fer-4* seedlings exposed to 3 days of ML (Figure 7A). However, strong Ca^2+^ signals were detected in the regions of abnormal chlorophyll autofluorescence signals in *fer-4* mutants exposed to 7 days of ML (Figure 7B and Supplementary Figure 13). In these regions, chlorophyll autofluorescence was not restricted to the chloroplast, but was spread outside of the cells, which was similar to the pattern observed in burst cells exposed to strong light (Kasahara et al., 2002). As chloroplasts contain high concentrations of Ca^2+^ and control cytoplasmic Ca^2+^ levels (Rocha and Vothknecht, 2012; Navazio et al., 2020), it seems that the disruption of cells and chloroplasts by photooxidative damage in *fer-4* mutants caused the loss of Ca^2+^ homeostasis, which resulted in the accumulation of Ca^2+^ in damaged cells. In addition, stimulation-induced cytoplasmic Ca^2+^ signals are mainly observed at the very early time points after the stimulation (Lee and Seo, 2021), but noticeable increase of Ca^2+^ signals were not observed up to 3 days after ML exposure. Therefore, we concluded that increased Ca^2+^ signals in *fer-4* mutants under ML are not the primary factor for the disrupted photoprotection, but the results of cell damage.

**FIGURE 8:**
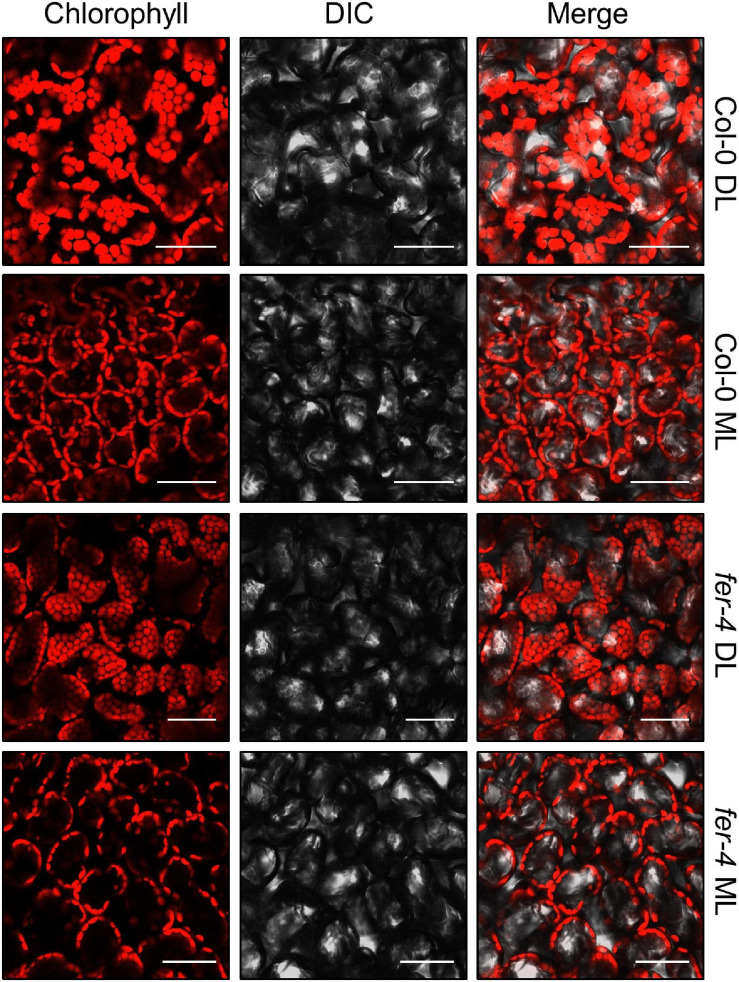
Chloroplast avoidance in the *fer-4* mutants under different light intensities. Six-day-old seedlings grown under DL were incubated in the dark for 16 h, and then exposed to DL or ML for 3 h. Adaxial side of the cotyledons was used for observing chlorophyll autofluorescence. Confocal microscope was used to analyze fluorescence. Size markers indicate 50 μm.

To avoid the exposure of chloroplasts to high-intensity light, chloroplasts move to the side walls of the cells, which is called chloroplast avoidance (Kagawa et al., 2001; Kasahara et al., 2002). Because FER is involved in photooxidative damage in chloroplasts, we observed chloroplast locations under different light intensities. However, chloroplasts in both Col-0 and *fer-4* seedlings similarly exhibited chloroplast avoidance (Figure 8), suggesting that FER is not involved in these responses.

### FER Accumulates Under ML

**FIGURE 9:**
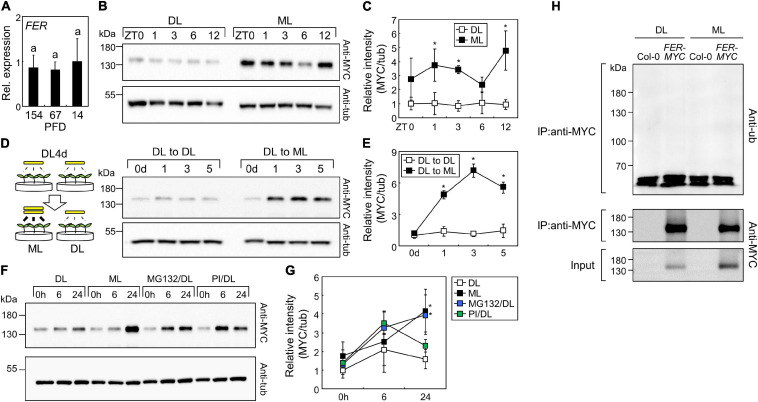
FERONIA (FER) proteins accumulate under ML. Whiskers indicate ± SD. **(A)** Expression of the *FER* gene under different light intensities. Seedlings were grown under different light intensities for 7 days. Whole seedlings were harvested at ZT3. *X*-axis numbers indicate PFD (μmol m^–2^ s^–1^). Biological triplicates were averaged. Letters indicate groups that are statistically significantly different from each other (*P* < 0.05, Tukey’s test). **(B,C)** Time-course analysis of FER protein abundance. *FER_*pro*_:FER-MYC* seedlings were grown under ML or DL for 7 days. Aerial parts of the seedlings were harvested at the indicated time points. Immunoblot analyses with anti-MYC and anti-a-tubulin (anti-tub) were performed. Representative images were displayed in panel **(B)**. Intensities of FER-MYC immunoblot bands were divided by those of tub bands **(C)**. Three replicates were averaged and statistically analyzed using Student’s *t*-test (*P < 0.05; difference from DL). **(D,E)** Effects of increasing light intensity on FER protein abundance. *FER_*pro*_:FER-MYC* seedlings were grown under DL for 4 days and then transferred to ML or left under DL for the indicated time periods. Aerial parts of the seedlings were harvested at ZT3. Representative images **(D)** and relative intensities of FER-MYC immunoblot bands **(E)** were displayed. Three replicates were averaged and statistically analyzed using Student’s *t*-test (*P < 0.05; difference from “DL to DL”). **(F,G)** Effects of MG132 and protease inhibitors (PI) on FER protein abundance. *FER_*pro*_:FER-MYC* seedlings were grown under DL for 6 days and then transferred to ML or left under DL with mock, MG132 (5 μM), or PI treatment for the indicated time periods. Aerial parts of the seedlings were harvested. Representative images **(F)** and relative intensities of FER-MYC immunoblot bands **(G)** were displayed. Three replicates were averaged and statistically analyzed using Student’s *t*-test (*P < 0.05; difference from DL). **(H)** Analysis of FER ubiquitination. The Col-0 and *FER_*pro*_:FER-MYC* (*FER-MYC*) seedlings were grown under DL or ML for 6 days and then subjected to MG132 treatment for 24 h. Whole seedlings were harvested after MG132 treatment. IP, immunoprecipitation; anti-ub, anti-ubiquitin.

Next, we analyzed FER abundance under different light intensities. The expression of *FER* was not affected by changes in light intensities (Figure 9A); thus, we examined FER protein abundance using *FER_*pro*_:FER-MYC* transgenic seedlings. Time-course analysis of 7-day-old seedlings showed that FER proteins accumulated more under ML than under DL (Figures 9B,C and Supplementary Figure 14). Then, we transferred the seedlings from DL to ML and analyzed their FER protein abundance, and found that ML treatment induced FER accumulation within 24 h (Figures 9D,E and Supplementary Figure 15). As protein abundance changed, but gene expression was unaffected by changes in light intensity, we analyzed translation efficiency of FER mRNA under DL and ML. However, analysis of total and polysome-associated FER mRNAs showed that translation efficiency was not altered by changing light intensity (Supplementary Figure 16). To determine the molecular mechanisms of protein regulation, we treated the seedlings grown under DL with 26S proteasome inhibitor MG132 and protease inhibitor cocktails. While protease inhibitor cocktails only slightly affected FER protein abundance, MG132 significantly increased FER protein stability under DL (Figures 9F,G and Supplementary igure 17). To examine the proteasome-mediated regulation of FER, we analyzed protein ubiquitination, which is a pre-requisite for proteasomal degradation (Vierstra, 2009). Immunoprecipitation of FER-MYC fusion proteins using the anti-MYC antibody showed that FER proteins were not ubiquitinated (Figure 9H and Supplementary Figure 18). These results indicate that the ubiquitin-proteasome system indirectly controls FER protein abundance in response to changing light intensity.

Altogether, we found that plants require protection against photooxidative damage not only under extremely high light but also under moderate light conditions, and that FER plays a pivotal role in their photoprotection. FER proteins accumulate under ML via indirect ubiquitination-proteasome pathways. Plasma membrane-localized FER alleviates excitation pressure on PSII and accumulation of ROS possibly by regulation of genes involved in stress responses, ROS metabolism, and photosynthesis.

## Discussion

### Plasma Membrane-Localized FER Regulates Photooxidative Stress

Our data showed that FER protein abundance is regulated by light intensity. High-intensity light generally produces excess excitation energy in the photosynthetic machinery; thus, chloroplasts are the main sites for sensing excess light (Mullineaux and Karpinski, 2002). However, FER is localized in the plasma membrane and its precise location is possibly important for its function in photoprotection, because disruption of FER localization by *llg1* mutation resulted in photobleaching (Li et al., 2015; Figure 6A). Regarding how excess light signals control FER abundance, we suspected that a possible mechanism for these responses is chloroplast retrograde signaling. ROS produced during photosynthesis in the chloroplast trigger retrograde signaling to the nucleus and regulate gene expression involved in stress responses (Maruta et al., 2012; Leister, 2019). It is also possible that altered redox states in the photosynthetic electron transport components as a consequence of excess light produce retrograde signaling. It has been reported that light-induced changes in redox states in the plastoquinone send retrograde signals to control alternative splicing in the nucleus (Petrillo et al., 2014). Although the detailed mechanisms of ROS- and redox change-induced chloroplast retrograde signaling remain unknown, it is possible that ML-induced chloroplast retrograde signals directly or indirectly affect plasma membrane-located FER. On the other hand, blue light receptor phototropins (PHOTs) are putative upstream regulators of FER. High light-induced chloroplast avoidance is mediated by PHOTs; thus, defective chloroplast movement in *phot* mutants triggers photobleaching and inhibition of photosynthetic ability under high light conditions (Kasahara et al., 2002). Screening of FER-interacting proteins under different light intensity is necessary for identifying the molecular mechanisms of FER function in photoprotection.

Based on our data, we concluded that FER regulates the expression of genes involved in stress responses and ROS metabolism under ML conditions (Figures 2B,D, 3). In addition, FER reduces ROS accumulation under the same conditions (Figure 5). While we observed these two different responses separately, it is possible that the FER-controlled gene expressions block photooxidative damage by scavenging ROS. The expression of stress genes is related to stress tolerance. For example, overexpression of the rice gene *OsAP21* in Arabidopsis induces salt and drought tolerance with high expression of *RD29B* (Jin et al., 2013). In addition, Arabidopsis PEROXISOME DEFECTIVE 2 (PED2)-deficient mutants exhibit reduced drought tolerance and decreased expression of stress genes, including *RD29B* (Shi et al., 2015). ROS content in *ped2* mutants is higher than that in wild type plants under drought conditions (Shi et al., 2015), implying that the expression of stress genes is related to the management of ROS content in plants. We also observed that the expression of *APX2* increased under ML and depended on FER (Figure 2B). APX2 plays a key role in antioxidant functions and modulates ROS homeostasis to protect chloroplasts (Wu et al., 2018). Along with the results of these studies, our results suggested that FER-mediated gene expressions might confer resistance to photooxidative damage by suppressing ROS accumulation under ML.

### Function of FER in Photoprotection

While the expression of stress genes was induced at 154 μmol m^–2^ s^–1^ in Col-0 seedlings, their expression did not increased at 67 μmol m^–2^ s^–1^ in either Col-0 or *fer-4* seedlings (Figure 2A). However, *fer-4* mutants exhibited reduced growth and chlorophyll content not only at 154 μmol m^–2^ s^–1^ but also at 67 μmol m^–2^ s^–1^ (Figure 1A), suggesting that the induction of stress genes is not the only role of FER. Because FER is a receptor-like kinase (Höfte, 2015), it is possible that FER phosphorylates the proteins related to photoprotection. Further studies on the interactions between light-responsive proteins and FER under different light intensities would be helpful to fully understand how plants manage light stress under different light conditions.

FERONIA is a plasma membrane-localized protein which senses cell wall integrity and peptide signals via its extracellular domain and sends downstream signals by protein phosphorylation through its intracellular kinase domain (Haruta et al., 2014; Höfte, 2015; Feng et al., 2018). FER affects gene expression that occurs in the nucleus; thus, there should be intermediating proteins that link FER with gene expression. Membrane-localized transcription factors are possible candidates for these responses because they are known to be activated under stress conditions and transported to the nucleus for the expression of target genes (Seo et al., 2008). In combination with the observations that excess light triggers ROS accumulation and changes membrane structure (Mullineaux and Karpinski, 2002; Das and Roychoudhury, 2014), ML-induced ROS may affect membrane structure, which can influence FER activity for stress responses. The possible scenario is that the activated FER proteins in the plasma membrane control membrane-localized transcription factors to regulate gene expressions related to photoprotection in the chloroplasts. Further studies on the role of each FER domain in photoprotection would provide important information on the detailed molecular mechanisms of FER-mediated photoprotection.

While FER-deficient mutant showed strong photobleaching phenotype at seedling stage, adult *fer-4* mutants did not show defects in chlorophyll content under ML (Figure 1C and Supplementary Figure 4). Instead, significant reduction of growth parameters was observed in this condition. Decrease of growth was also observed under DL, but the magnitude of difference was much smaller than that under ML. The growth inhibition of FER-deficient mutant has been reported previously (Guo et al., 2009; Deslauriers and Larsen, 2010), but the detailed molecular mechanisms and related signaling pathways were poorly identified. It is possible that increased ethylene signals in *fer* mutants is one of the reasons, but it cannot fully explain reduced growth phenotype because inhibition of ethylene signaling in *fer* mutants did not recover growth retardation (Deslauriers and Larsen, 2010). Our data suggest that growth inhibition of *fer-4* mutants was due to reduced ability of photoprotection even under moderate light conditions. However, it remains unknown why FER-deficient mutant did not exhibit photobleaching phenotype at adult stage. Because abiotic stress resistance is different at different plant ages (Rankenberg et al., 2021), one possibility is that other FER-independent photoprotection pathways are activated at adult stage, while they are not sufficient to fully complement loss of FER functions.

FERONIA is highly conserved in land plants including Arabidopsis, rice, poplar, and moss (Escobar-Restrepo et al., 2007; Li et al., 2016b), which are directly exposed to increased intensity of light compared to aquatic plants. Therefore, it is possible that plants need to develop photoprotection systems for adaptation to non-aquatic environment. In support of this idea, submerged aquatic freshwater plant species are highly susceptible to increasing light intensity. Exposure of these aquatic plant species to moderate light (100 μmol m^–2^ s^–1^) results in rapid decrease of maximum PSII quantum yield (Hussner et al., 2010). In combination with our data, FER-mediated photoprotection might be a key factor to maintain photosystems after land plant evolution.

### Putative Role of FER in Sun and Shade Plants

Previous studies have mainly focused on plant responses to high light with the intensity of 500–1,500 μmol m^–2^ s^–1^. Our findings showed that *fer-4* mutants exhibited chlorophyll loss and cell death at 67–154 μmol m^–2^ s^–1^, which is a relatively low light intensity. These results suggested that plants need to manage light stress even under moderate light intensity through the function of FER. In nature, different plant species have different levels of light sensitivity. Sun and shade plants have developed different photosynthetic and photoprotective mechanisms; thus, they require different light intensities for optimal growth (Mathur et al., 2018). For example, *Panax ginseng* is a shade plant which exhibits impaired photosynthetic machinery functions and ROS accumulation in the chloroplasts under high light intensities that are normal for sun plants (Powles, 1984; Jung et al., 2020). Based on the role of FER in Arabidopsis, we anticipated that different functions of FER can determine if plants are sun or shade plants. A combination of phylogenetic analysis and comparison of molecular functions among FER homologs in sun and shade plants would provide clues for identifying evolutionary events in these plants.

## Data Availability Statement

The original contributions presented in the study are included in the article/Supplementary Material, further inquiries can be directed to the corresponding author. The raw and processed RNA sequencing data have been deposited in NCBI’s Gene Expression Omnibus and are accessible through GEO Series accession number GSE172298.

## Author Contributions

SYS and H-JL conceived and designed the experiments, performed the trypan blue and ROS staining, and analyzed other experiments with help of K-BM. SYS analyzed the phenotype and chlorophyll content of seedlings. SYS and J-SP analyzed the gene expressions. H-BP and H-JL performed the immunoblot assays. H-SK, J-HJ, and HSC provided experimental equipment and scientific discussions. H-JL prepared the manuscript with the contributions of SYS. All authors contributed to the article and approved the submitted version.

## Conflict of Interest

The authors declare that the research was conducted in the absence of any commercial or financial relationships that could be construed as a potential conflict of interest.

## Footnotes

1 https://www.arabidopsis.org

2 https://imagej.nih.gov/ij/

## References

[B1] Agati G. Brunetti C. Ferdinando M. D. Ferrini F. Pollastri S. Tattini M. (2013). Functional roles of flavonoids in photoprotection: new evidence, lessons from the past. *Plant Physiol. Biochem.* 72 35–45. 10.1016/j.plaphy.2013.03.014 23583204

[B2] Bergonci T. Ribeiro B. Ceciliato P. H. O. Guerrero-Abad J. C. Silva-Filho M. C. Moura D. S. (2014). Arabidopsis thaliana RALF1 opposes brassinosteroid effects on root cell elongation and lateral root formation. *J. Exp. Bot.* 65 2219–2230. 10.1093/jxb/eru099 24620000 PMC3991750

[B3] Bouquin T. Meier C. Foster R. Nielsen M. E. Mundy J. (2001). Control of specific gene expression by gibberellin and brassinosteroid. *Plant Physiol.* 127 450–458. 10.1104/pp.010173 11598220 PMC125081

[B4] Chen J. Yu F. Liu Y. Du C. Li X. Zhu S. (2016). FERONIA interacts with ABI2-type phosphatases to facilitate signaling cross-talk between abscisic acid and RALF peptide in *Arabidopsis*. *Proc. Natl. Acad. Sci. U.S.A.* 113 E5519–E5527. 10.1073/pnas.1608449113 27566404 PMC5027425

[B5] Chen L. T. Luo M. Wang Y. Y. Wu K. (2010). Involvement of *Arabidopsis* histone deacetylase HDA6 in ABA and salt stress response. *J. Exp. Bot.* 61 3345–3353. 10.1093/jxb/erq154 20519338 PMC2905197

[B6] Das K. Roychoudhury A. (2014). Reactive oxygen species (ROS) and response of antioxidants as ROS-scavengers during environmental stress in plants. *Front. Environ. Sci.* 2:53. 10.3389/fenvs.2014.00053

[B7] Daudi A. O’Brien J. A. (2012). Detection of hydrogen peroxide by DAB staining in *Arabidopsis* leaves. *Bio Protoc.* 2:e263. 10.21769/BioProtoc.263 27390754 PMC4932902

[B8] Deslauriers S. D. Larsen P. B. (2010). FERONIA is a key modulator of brassinosteroid and ethylene responsiveness in *Arabidopsis* hypocotyls. *Mol. Plant* 3 626–640. 10.1093/mp/ssq015 20400488

[B9] Dong Q. Zhang Z. Liu Y. Tao L. Z. Liu H. (2019). FERONIA regulates auxin-mediated lateral root development and primary root gravitropism. *FEBS Lett.* 593 97–106. 10.1002/1873-3468.13292 30417333

[B10] Duan Q. Kita D. Johnson E. A. Aggarwal M. Gates L. Wu H. M. (2014). Reactive oxygen species mediate pollen tube rupture to release sperm for fertilization in *Arabidopsis*. *Nat. Commun.* 5:3129. 10.1038/ncomms4129 24451849

[B11] Duan Q. Kita D. Li C. Cheung A. Y. Wu H. M. (2010). FERONIA receptor-like kinase regulates RHO GTPase signaling of root hair development. *Proc. Natl. Acad. Sci. U.S.A.* 107 17821–17826. 10.1073/pnas.1005366107 20876100 PMC2955125

[B12] Dunand C. Crèvecoeur M. Penel C. (2007). Distribution of superoxide and hydrogen peroxide in *Arabidopsis* root and their influence on root development: possible interaction with peroxidases. *New Phytol.* 174 332–341. 10.1111/j.1469-8137.2007.01995.x 17388896

[B13] Escobar-Restrepo J. M. Huck N. Kessler S. Gagliardini V. Gheyselinck J. Yang W. C. (2007). The FERONIA receptor-like kinase mediates male-female interactions during pollen tube reception. *Science* 317 656–660. 10.1126/science.1143562 17673660

[B14] Feng W. Kita D. Peaucelle A. Cartwright H. N. Doan V. Duan Q. (2018). The FERONIA receptor kinase maintains cell-wall integrity during salt stress through Ca^2+^ signaling. *Curr. Biol.* 28 666–675. 10.1016/j.cub.2018.01.023 29456142 PMC5894116

[B15] Flors C. Fryer M. J. Waring J. Reeder B. Bechtold U. Mullineaux P. M. (2006). Imaging the production of singlet oxygen in vivo using a new fluorescent sensor, singlet oxygen sensor green. *J. Exp. Bot.* 57 1725–1734. 10.1093/jxb/erj181 16595576

[B16] Galvez-Valdivieso G. Fryer M. J. Lawson T. Slattery K. Truman W. Smirnoff N. (2009). The high light response in *Arabidopsis* involves ABA signaling between vascular and bundle sheath cells. *Plant Cell* 21 2143–2162. 10.1105/tpc.108.061507 19638476 PMC2729609

[B17] Garcia-Molina A. Leister D. (2020). Accelerated relaxation of photoprotection impairs biomass accumulation in *Arabidopsis*. *Nat. Plants* 6 9–12. 10.1038/s41477-019-0572-z 31907400

[B18] Guo H. Li L. Ye H. Yu X. Algreen A. Yin Y. (2009). Three related receptor-like kinases are required for optimal cell elongation in *Arabidopsis thaliana*. *Proc. Natl. Acad. Sci. U.S.A.* 106 7648–7653. 10.1073/pnas.0812346106 19383785 PMC2678668

[B19] Guo H. Nolan T. M. Song G. Liu S. Xie Z. Chen J. (2018). FERONIA receptor kinase contributes to plant immunity by suppressing jasmonic acid signaling in Arabidopsis thaliana. *Curr. Biol.* 28 3316–3324. 10.1016/j.cub.2018.07.078 30270181

[B20] Ha J. H. Lee H. J. Jung J. H. Park C. M. (2017). Thermo-induced maintenance of photo-oxidoreductases underlies plant autotrophic development. *Dev. Cell* 41 170–179. 10.1016/j.devcel.2017.03.005 28392197

[B21] Haruta M. Sabat G. Stecker K. Minkoff B. B. Sussman M. R. (2014). A peptide hormone and its receptor protein kinase regulate plant cell expansion. *Science* 343 408–411. 10.1126/science.1244454 24458638 PMC4672726

[B22] Havaux M. Eymery F. Porfirova S. Rey P. Dörmann P. (2005). Vitamin E protects against photoinhibition and photooxidative stress in *Arabidopsis thaliana*. *Plant Cell* 17 3451–3469. 10.1105/tpc.105.037036 16258032 PMC1315381

[B23] Hideg E. Kálai T. Hideg K. Vass I. (1998). Photoinhibition of photosynthesis in vivo results in singlet oxygen production detection via nitroxide-induced fluorescence quenching in broad bean leaves. *Biochemistry* 37 11405–11411. 9708975 10.1021/bi972890+

[B24] Hideg E. Spetea C. Vass I. (1994). Singlet oxygen production in thylakoid membranes during photoinhibition as detected by EPR spectroscopy. *Photosynth. Res.* 39 191–199. 10.1007/BF00029386 24311071

[B25] Höfte H. (2015). The yin and yang of cell wall integrity control: brassinosteroid and FERONIA signaling. *Plant Cell Physiol.* 56 224–231. 10.1093/pcp/pcu182 25481004

[B26] Huang G. Q. Li E. Ge F. R. Li S. Wang Q. Zhang C. Q. (2013). *Arabidopsis* RopGEF4 and RopGEF10 are important for FERONIA-mediated developmental but not environmental regulation of root hair growth. *New Phytol.* 200 1089–1101. 10.1111/nph.12432 23915272

[B27] Hubbart S. Smillie I. R. A. Heatley M. Swarup R. Foo C. C. Zhao L. (2018). Enhanced thylakoid photoprotection can increase yield and canopy radiation use efficiency in rice. *Commun. Biol.* 1:22. 10.1038/s42003-018-0026-6 30271909 PMC6123638

[B28] Hussner A. Hoelken H. P. Jahns P. (2010). Low light acclimated submerged freshwater plants show a pronounced sensitivity to increasing irradiances. *Aquat. Bot.* 93 17–24. 10.1016/j.aquabot.2010.02.003

[B29] Jin X. Xue Y. Wang R. Xu R. Bian L. Zhu B. (2013). Transcription factor *OsAP21* gene increases salt/drought tolerance in transgenic *Arabidopsis thaliana*. *Mol. Biol. Rep.* 40 1743–1752. 10.1007/s11033-012-2228-1 23104474

[B30] Jung J. H. Kim H. Y. Kim H. S. Jung S. H. (2020). Transcriptome analysis of Panax ginseng response to high light stress. *J. Ginseng Res.* 44 312–320. 10.1016/j.jgr.2018.12.009 32148414 PMC7031748

[B31] Kagawa T. Sakai T. Suetsugu N. Oikawa K. Ishiguro S. Kato T. (2001). *Arabidopsis* NPL1: a phototropin homolog controlling the chloroplast high-light avoidance response. *Science* 291 2138–2141. 10.1126/science.291.5511.2138 11251116

[B32] Kasahara M. Kagawa T. Oikawa K. Suetsugu N. Miyao M. Wada M. (2002). Chloroplast avoidance movement reduces photodamage in plants. *Nature* 420 829–832. 10.1038/nature01213 12490952

[B33] Kato K. Shinoda T. Nagao R. Akimoto S. Suzuki T. Dohmae N. (2020). Structural basis for the adaptation and function of chlorophyll f in photosystem I. *Nat. Commun.* 11:238. 10.1038/s41467-019-13898-5 31932639 PMC6957486

[B34] Khanal N. Bray G. E. Grisnich A. Moffatt B. A. Gray G. R. (2017). Differential mechanisms of photosynthetic acclimation to light and low temperature in *Arabidopsis* and the extremophile Eutrema salsugineum. *Plants* 6:32. 10.3390/plants6030032 28792470 PMC5620588

[B35] Kim J. H. Lee H. J. Jung J. H. Lee S. Park C. M. (2017). HOS1 facilitates the phytochrome B-mediated inhibition of PIF4 function during hypocotyl growth in *Arabidopsis*. *Mol. Plant* 10 274–284. 10.1016/j.molp.2016.11.009 27890635

[B36] Ksas B. Becuwe N. Chevalier A. Havaux M. (2015). Plant tolerance to excess light energy and photooxidative damage relies on plastoquinone biosynthesis. *Sci. Rep.* 5:10919. 10.1038/srep10919 26039552 PMC4454199

[B37] Kudla J. Becker D. Grill E. Hedrich R. Hippler M. Kummer U. (2018). Advances and current challenges in calcium signaling. *New Phytol.* 218 414–431. 10.1111/nph.14966 29332310

[B38] Laloi C. Havaux M. (2015). Key players of singlet oxygen-induced cell death in plants. *Front. Plant Sci.* 6:39. 10.3389/fpls.2015.00039 25699067 PMC4316694

[B39] Lecampion C. Floris M. Fantino J. R. Robaglia C. Laloi C. (2016). An easy method for plant polysome profiling. *J. Vis. Exp.* 114:54231. 10.3791/54231 27684295 PMC5091964

[B40] Lecourieux D. Ranjeva R. Pugin A. (2006). Calcium in plant defence-signalling pathways. *New Phytol.* 171 249–269. 10.1111/j.1469-8137.2006.01777.x 16866934

[B41] Lee H. J. Seo P. J. (2021). Ca^2+^talyzing initial responses to environmental stresses. in press, *Trends Plant Sci*. 10.1016/j.tplants.2021.02.007 33706981

[B42] Lee H. J. Kim H. S. Park J. M. Cho H. S. Jeon J. H. (2020). PIN-mediated polar auxin transport facilitates root-obstacle avoidance. *New Phytol.* 225 1285–1296. 10.1111/nph.16076 31336402

[B43] Leister D. (2019). Piecing the puzzle together: the central role of reactive oxygen species and redox hubs in chloroplast retrograde signaling. *Antioxid. Redox Signal.* 30 1206–1219. 10.1089/ars.2017.7392 29092621

[B44] Léon-Kloosterziel K. M. Gil M. A. Ruijs G. J. Jacobsen S. E. Olszewski N. E. Schwartz S. H. (1996). Isolation and characterization of abscisic acid-deficient Arabidopsis mutants at two new loci. *Plant J.* 10 655–661. 10.1046/j.1365-313x.1996.10040655.x 8893542

[B45] Li C. Wang L. Cui Y. He L. Qi Y. Zhang J. (2016b). Two FERONIA-like receptor (FLR) genes are required to maintain architecture, fertility, and seed yield in rice. *Mol. Breeding* 36:151. 10.1007/s11032-016-0580-x

[B46] Li C. Wu H. M. Cheung A. Y. (2016a). FERONIA and her pals: functions and mechanisms. *Plant Physiol.* 171 2379–2392. 10.1104/pp.16.00667 27342308 PMC4972288

[B47] Li C. Yeh F. L. Cheung A. Y. Duan Q. Kita D. Liu M. C. (2015). Glycosylphosphatidylinositol-anchored proteins as chaperones and co-receptors for FERONIA receptor kinase signaling in *Arabidopsis*. *eLife* 4:e06587. 10.7554/eLife.06587 26052747 PMC4458842

[B48] Li Z. Waadt R. Schroeder J. I. (2016c). Release of GTP exchange factor mediated down-regulation of abscisic acid signal transduction through ABA-induced rapid degradation of RopGEFs. *PLoS Biol.* 14:e1002461. 10.1371/journal.pbio.1002461 27192441 PMC4871701

[B49] Logan B. A. Kornyeyev D. Hardison J. Holaday A. S. (2006). The role of antioxidant enzymes in photoprotection. *Photosynth. Res.* 88 119–132. 10.1007/s11120-006-9043-2 16622785

[B50] Mansfield S. G. Briarty L. G. (1996). The dynamics of seedling and cotyledon cell development in *Arabidopsis thaliana* during reserve mobilization. *Int. J. Plant Sci.* 157 280–295. 10.1086/297347

[B51] Maruta T. Noshi M. Tanouchi A. Tamoi M. Yabuta Y. Yoshimura K. (2012). H_2_O_2_-triggered retrograde signaling from chloroplasts to nucleus plays specific role in response to stress. *J. Biol. Chem.* 287 11717–11729. 10.1074/jbc.M111.292847 22334687 PMC3320920

[B52] Masachis S. Segorbe D. Turrà D. Leon-Ruiz M. Fürst U. Ghalid M. E. (2016). A fungal pathogen secretes plant alkalinizing peptides to increase infection. *Nat. Microbiol.* 1:16043. 10.1038/nmicrobiol.2016.43 27572834

[B53] Mathur S. Jain L. Jajoo A. (2018). Photosynthetic efficiency in sun and shade plants. *Photosynthetica* 56 354–365. 10.1007/BF00377346 28310415

[B54] Maxwell K. Johnson G. N. (2000). Chlorophyll fluorescence—a practical guide. *J. Exp. Bot.* 51 659–668. 10.1093/jxb/51.345.659 10938857

[B55] Mikko T. Mirva P. Marjaana S. Sari S. Paula M. Julia V. (2006). State transitions revisited-a buffering system for dynamic low light acclimation of *Arabidopsis*. *Plant Mol. Biol.* 62 779–793. 10.1007/s11103-006-9044-8 16897465

[B56] Msanne J. Lin J. Stone J. M. Awada T. (2011). Characterization of abiotic stress-responsive *Arabidopsis thaliana RD29A* and *RD29B* genes and evaluation of transgenes. *Planta* 234 97–107. 10.1007/s00425-011-1387-y 21374086

[B57] Mullineaux P. Karpinski S. (2002). Signal transduction in response to excess light: getting out of the chloroplast. *Curr. Opin. Plant Biol.* 5 43–48. 10.1016/s1369-5266(01)00226-6 11788307

[B58] Murchie E. H. Niyogi K. K. (2011). Manipulation of photoprotection to improve plant photosynthesis. *Plant Physiol.* 155 86–92. 10.1104/pp.110.168831 21084435 PMC3075776

[B59] Navazio L. Formentin E. Cendron L. Szabò I. (2020). Chloroplast calcium signaling in the spotlight. *Front. Plant Sci.* 11:186. 10.3389/fpls.2020.00186 32226434 PMC7081724

[B60] Pascal A. A. Liu Z. Broess K. van Oort B. van Amerongen H. Wang C. (2005). Molecular basis of photoprotection and control of photosynthetic light-harvesting. *Nature* 436 134–137. 10.1038/nature03795 16001075

[B61] Petrillo E. Herz M. A. G. Fuchs A. Reifer D. Fuller J. Yanovsky M. J. (2014). A chloroplast retrograde signal regulates nuclear alternative splicing. *Science* 344 427–430. 10.1126/science.1250322 24763593 PMC4382720

[B62] Porra R. J. Thompson W. A. Kriedemann P. E. (1989). Determination of accurate extinction coefficients and simultaneous equations for assaying chlorophylls a and b extracted with four different solvents: verification of the concentration of chlorophyll standards by atomic absorption spectroscopy. *Biochim. Biophys. Acta* 975 384–394. 10.1016/S0005-2728(89)80347-0

[B63] Powles S. B. (1984). Photoinhibition of photosynthesis induced by visible light. *Annu. Rev. Plant Physiol.* 35 15–44. 10.1146/annurev.pp.35.060184.000311

[B64] Rankenberg T. Geldhof B. van Veen H. Holsteens K. Van de Poel B. Sasidharan R. (2021). Age-dependent abiotic stress resilience in plants. *Trends Plant Sci*. 26 692–705. 10.1016/j.tplants.2020.12.016 33509699

[B65] Rocha A. G. Vothknecht U. C. (2012). The role of calcium in chloroplasts-an intriguing and unresolved puzzle. *Protoplasma* 249 957–966. 10.1007/s00709-011-0373-3 22227834

[B66] Rossel J. B. Wilson I. W. Pogson B. J. (2002). Global changes in gene expression in response to high light in *Arabidopsis*. *Plant Physiol.* 130 1109–1120. 10.1104/pp.005595 12427978 PMC166632

[B67] Rossel J. B. Wilson P. B. Hussain D. Woo N. S. Gordon M. J. Mewett O. P. (2007). Systemic and intracellular responses to photooxidative stress in *Arabidopsis*. *Plant Cell* 19 4091–4110. 10.1105/tpc.106.045898 18156220 PMC2217654

[B68] Seo P. J. Kim S. G. Park C. M. (2008). Membrane-bound transcription factors in plants. *Trends Plant Sci.* 13 550–556. 10.1016/j.tplants.2008.06.008 18722803

[B69] Shi H. Ye T. Yang F. Chan Z. (2015). *Arabidopsis* PED2 positively modulates plant drought stress resistance. *J. Integr. Plant Biol.* 57 796–806. 10.1111/jipb.12330 25588806

[B70] Shih H. W. Miller N. D. Dai C. Spalding E. P. Monshausen G. B. (2014). The receptor-like kinase FERONIA is required for mechanical signal transduction in *Arabidopsis* seedlings. *Curr. Biol.* 24 1887–1892. 10.1016/j.cub.2014.06.064 25127214

[B71] Takahashi S. Badger M. R. (2011). Photoprotection in plants: a new light on photosystem II damage. *Trends Plant Sci.* 16 53–60. 10.1016/j.tplants.2010.10.001 21050798

[B72] Vierstra R. D. (2009). The ubiquitin-26S proteasome system at the nexus of plant biology. *Nat. Rev. Mol. Cell Biol.* 10 385–397. 10.1038/nrm2688 19424292

[B73] Wang C. Xu W. Jin H. Zhang T. Lai J. Zhou X. (2016). A putative chloroplast-localized Ca^2+^/H^+^ antiporter CCHA1 is involved in calcium and pH homeostasis and required for PSII function in *Arabidopsis*. *Mol. Plant* 9 1183–1196. 10.1016/j.molp.2016.05.015 27302341

[B74] Wang L. Yang T. Lin Q. Wang B. Li X. Luan S. (2020). Receptor kinase FERONIA regulates flowering time in *Arabidopsis*. *BMC Plant Biol.* 20:26. 10.1186/s12870-019-2223-y 31948398 PMC6966814

[B75] Woodson J. D. (2019). Chloroplast stress signals: regulation of cellular degradation and chloroplast turnover. *Curr. Opin. Plant Biol.* 52 30–37. 10.1016/j.pbi.2019.06.005 31442733

[B76] Wu B. Li L. Qiu T. Zhang X. Cui S. (2018). Cytosolic APX2 is a pleiotropic protein involved in H_2_O_2_ homeostasis, chloroplast protection, plant architecture and fertility maintenance. *Plant Cell Rep.* 37 833–848. 10.1007/s00299-018-2272-y 29549445

[B77] Yang T. Wang L. Li C. Liu Y. Zhu S. Qi Y. (2015). Receptor protein kinase FERONIA controls leaf starch accumulation by interacting with glyceraldehyde-3-phosphate dehydrogenase. *Biochem. Biophys. Res. Commun.* 465 77–82. 10.1016/j.bbrc.2015.07.132 26232644

[B78] Yu F. Qian L. Nibau C. Duan Q. Kita D. Levasseur K. (2012). FERONIA receptor kinase pathway suppresses abscisic acid signaling in *Arabidopsis* by activating ABI2 phosphatase. *Proc. Natl. Acad. Sci. U.S.A.* 109 14693–14698. 10.1073/pnas.1212547109 22908257 PMC3437822

[B79] Zhang X. Henriques R. Lin S. S. Niu Q. W. Chua N. H. (2006). Agrobacterium-mediated transformation of *Arabidopsis thaliana* using the floral dip method. *Nat. Protoc.* 1 641–646. 10.1038/nprot.2006.97 17406292

[B80] Zhu S. Estévez J. M. Liao H. Zhu Y. Yang Y. Li C. (2020). The RALF1-FERONIA complex phosphorylates eIF4E1 to promote protein synthesis and polar root hair growth. *Mol. Plant* 13 698–716. 10.1016/j.molp.2019.12.014 31904511

