# Supplementary material for: FERONIA Confers Resistance to Photooxidative Stress in Arabidopsis
Source: Front Plant Sci. 2021 Jul 15;12:714938. doi: 10.3389/fpls.2021.714938 (PMC8320354; doi:10.3389/fpls.2021.714938)
Supplement: Supplementary file 2 [file Data_Sheet_1.PDF]

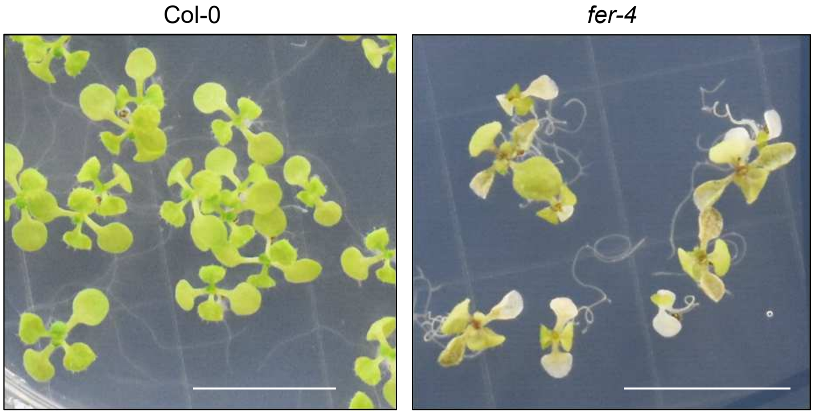

Supplementary Figure 1. Enlarged view of Col-0 and *fer-4* seedlings under ML.
Seedlings were grown on Murashige and Skoog-agar (MS-agar) plates for nine days under ML (PFD = 154 μmol m^{-2} s^{-1}). Scale bars indicate 1 cm.

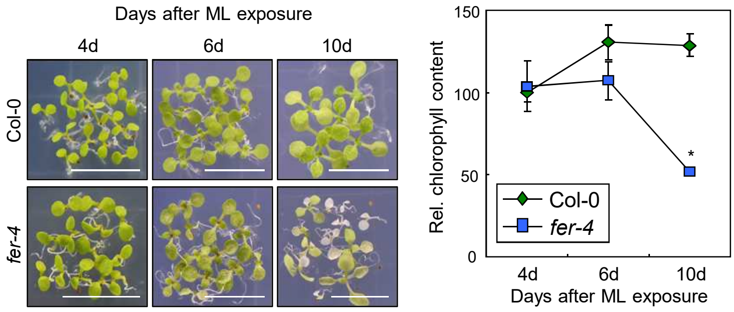

Supplementary Figure 2. Chlorophyll content after ML exposure.
Col-0 and *fer-4* seedlings were grown on MS-agar plates under long-day conditions (16 h light/8 h dark) under ML. Seedlings were photographed at the indicated time points (left panel). Whole seedlings were harvested and relative chlorophyll content was measured (right panel). Whiskers indicate ± SD. Biological triplicates were averaged and statistically analyzed using Student's *t*-test (*, $P < 0.01$; difference from Col-0). Scale bars indicate 1 cm.

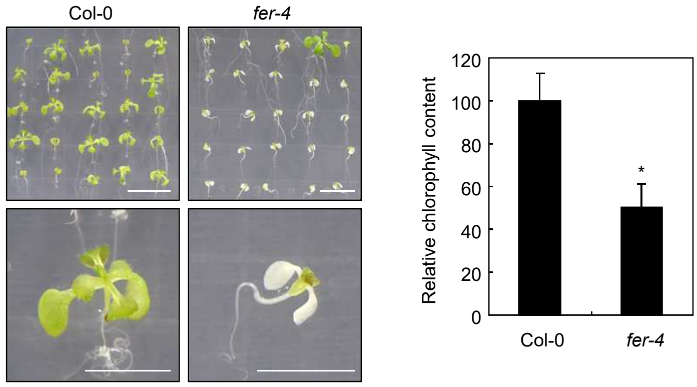

Supplementary Figure 3. Chlorophyll content under short-day conditions.
Col-0 and *fer-4* seedlings were grown on MS-agar plates for 17 days under short-day conditions (8 h light/16 h dark) at ML. Seedlings were photographed (left panel). Lower panel shows the enlarged view of the representative seedlings. Aerial parts of the seedlings were harvested and relative chlorophyll content was measured (right panel). Whiskers indicate standard deviation of the mean (SD). Biological triplicates were averaged and statistically analyzed using Student's *t*-test (*, $P < 0.01$; difference from Col-0). Scale bars indicate 1 cm. Scale bars in the enlarged view indicate 0.5 cm.

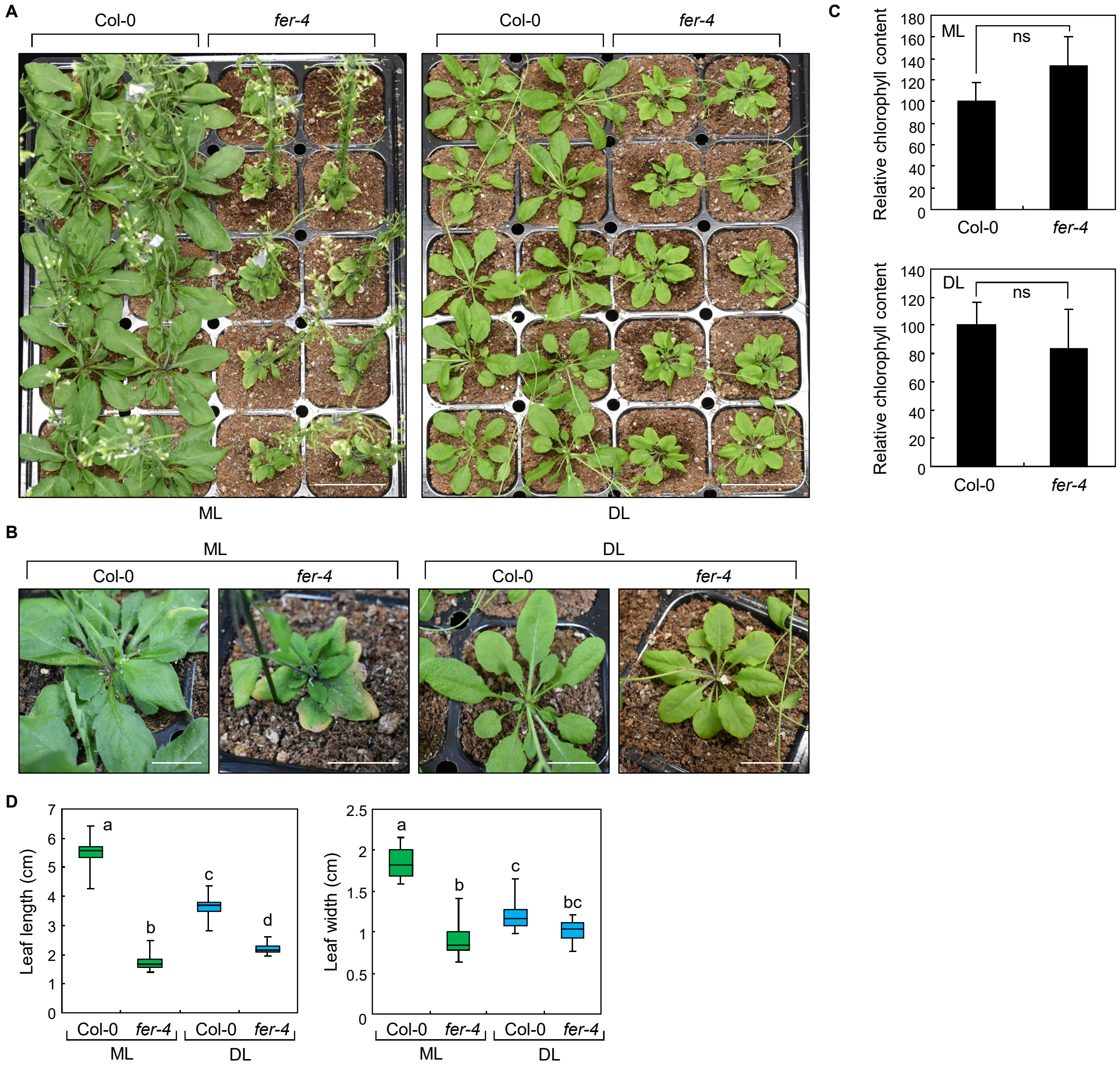

Supplementary Figure 4. Exposure of 3-week-old plants grown under DL to different light intensities. **(A)** Col-0 and *fer-4* plants were grown on soil for 3 weeks under DL (14 µmol m^{-2} s^{-1}) and then transferred to ML or left under DL for additional 3 weeks. Plants were grown under long-day conditions. Size markers indicate 5 cm.

(B) Enlarged representative images of plants grown under each condition. Size markers indicate 2 cm.

(C) Chlorophyll content in Col-0 and *fer-4* leaves. Plants were grown as described in (a). The 8th or 9th leaves were harvested to analyze chlorophyll content. Four biological replicates were averaged and statistically analyzed using Student's *t*-test (*, $P < 0.05$; difference from Col-0). ns, not significant. Whiskers indicate SD.

(D) Measurement of plant size. Col-0 and *fer-4* plants grown as described in **(A)** were used. Length and width of 7th rosette leaves were measured. Letters indicate groups that are statistically significantly different from each other (n = 14−15; $P < 0.01$, Tukey's test).

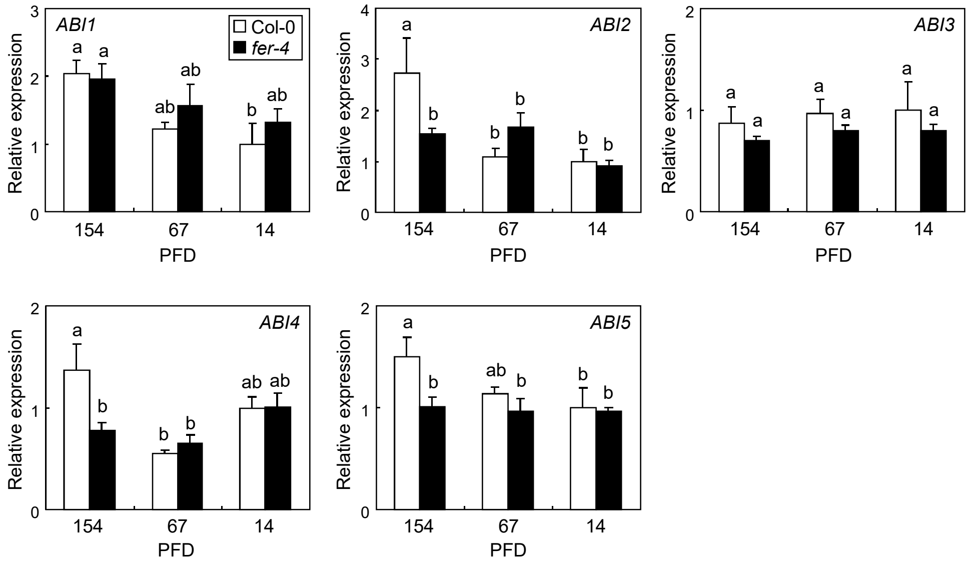

Supplementary Figure 5. Expression of *ABI* genes in *fer-4* mutants under different light intensities. The Col-0 and *fer-4* seedlings were grown on MS-agar plates at different light intensities for seven days under long-day conditions. Whole seedlings were harvested at zeitgeber time (ZT) 3 for total RNA extraction. X-axis numbers indicate PFD (μmol m^{-2} s^{-1}). Biological triplicates were averaged. Letters indicate groups that are statistically significantly different from each other ($P < 0.05$, Tukey's test). Whiskers indicate SD.

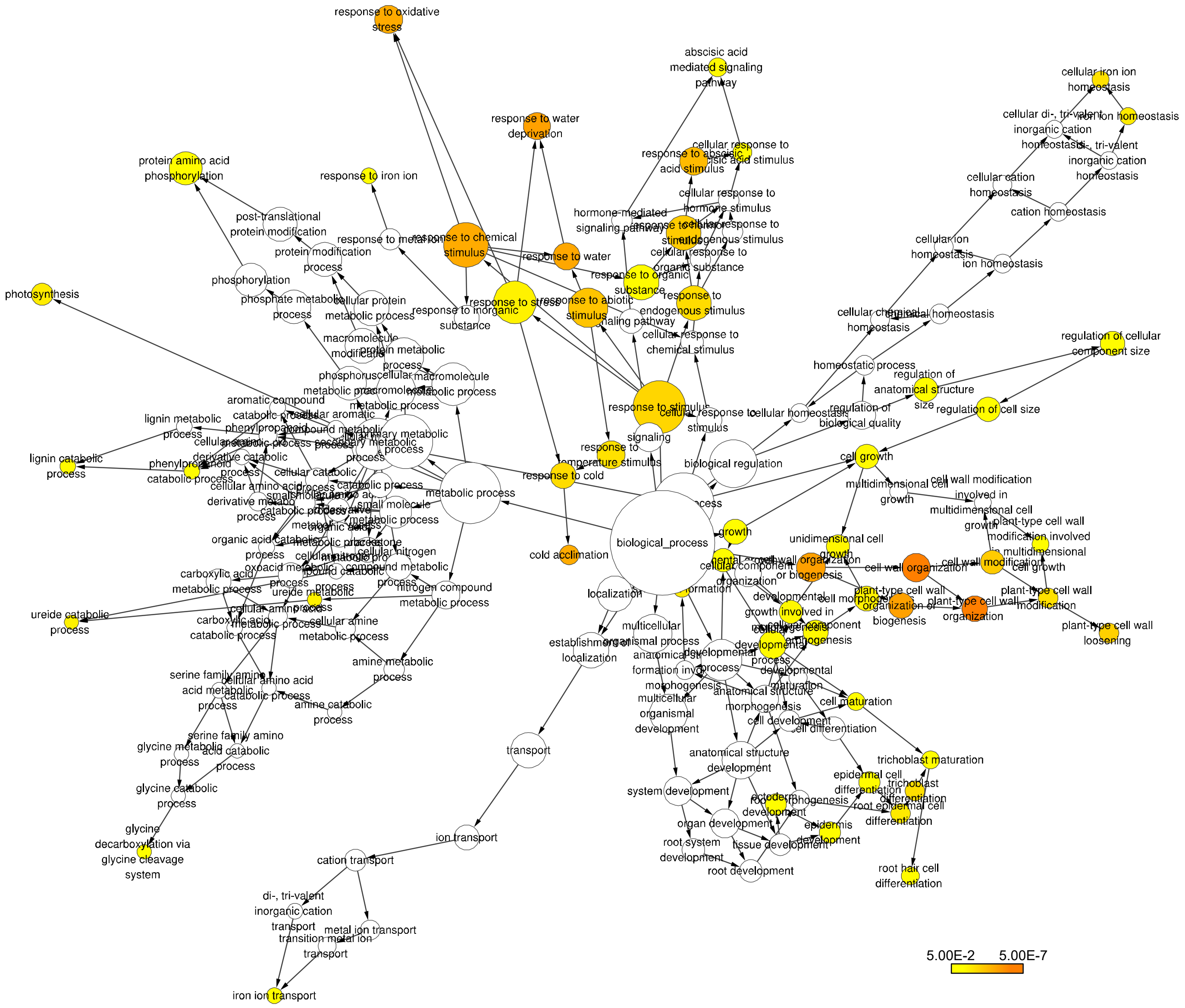

Supplementary Figure 6. Analysis of gene ontology (GO) biological process using down-regulated genes in *fer-4* mutants.

The Col-0 and *fer-4* seedlings were grown on MS-agar plates at ML for seven days under long-day conditions. Whole seedlings were harvested at ZT3 for total RNA extraction. RNA-sequencing was performed and differentially regulated genes were analyzed by comparing Col-0 and *fer-4*. GO biological process analysis was performed using down-regulated genes in *fer-4*. Colored nodes indicate significantly overrepresented GO terms (Benjamini-Hochberg-corrected $P < 0.05$). Scale bars indicate *P*-values.

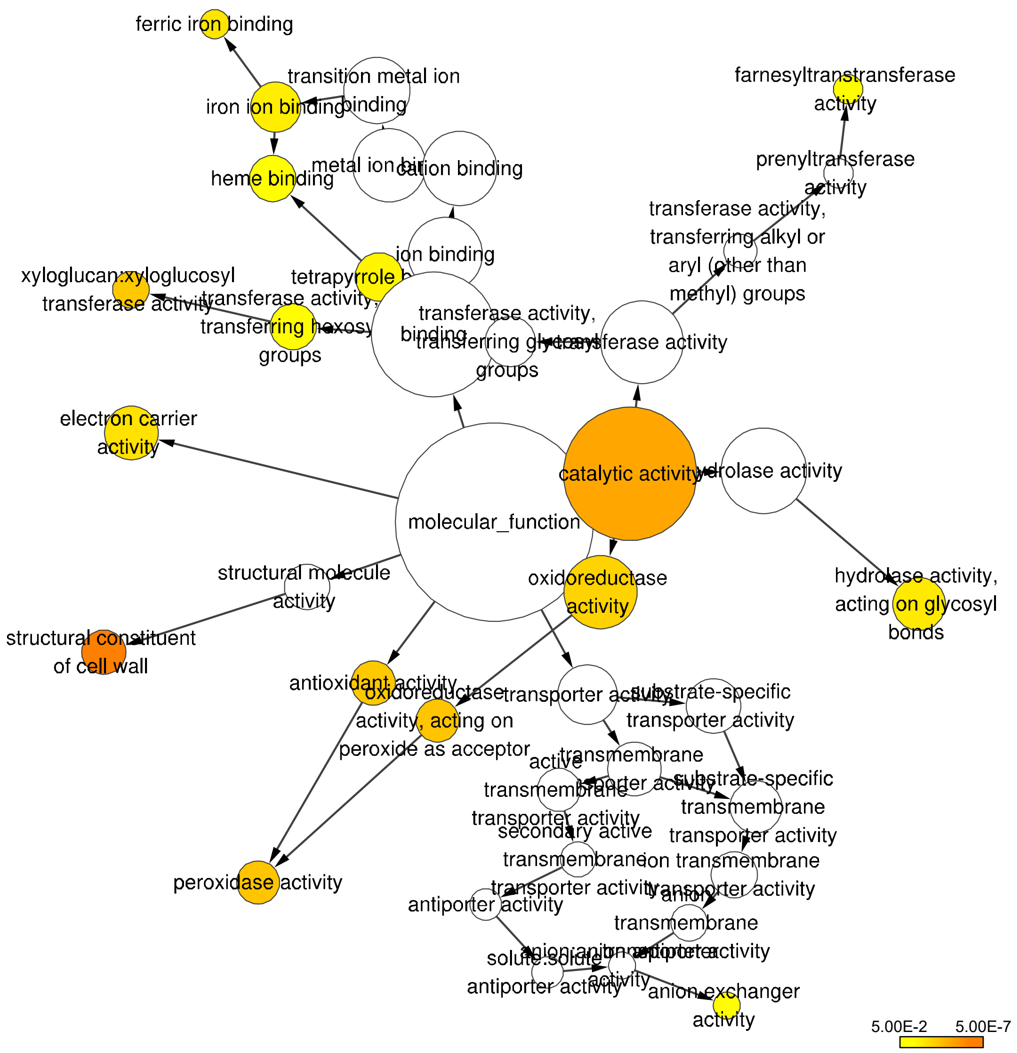

Supplementary Figure 7. Analysis of GO molecular function using down-regulated genes in *fer-4* mutants.

RNA-sequencing was performed and differentially regulated genes were analyzed as described in **Supplementary Figure 6**. GO molecular function analysis was performed using down-regulated genes in *fer-4*. Colored nodes indicate significantly overrepresented GO terms (Benjamini-Hochberg-corrected $P < 0.05$). Scale bars indicate P-values.

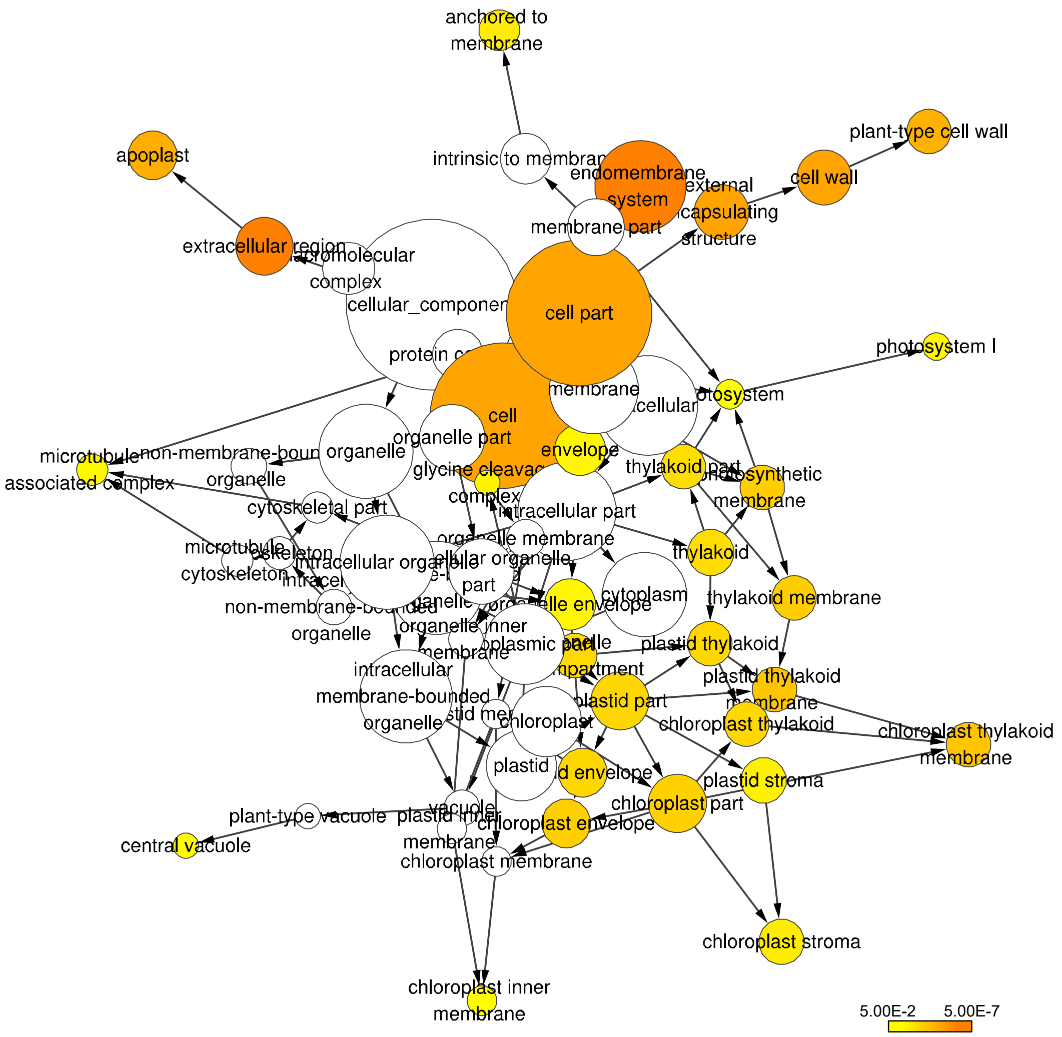

Supplementary Figure 8. Analysis of GO cellular component using down-regulated genes in *fer-4* mutants.

RNA-sequencing was performed and differentially regulated genes were analyzed as described in **Supplementary Figure 6**. GO cellular component analysis was performed using down-regulated genes in *fer-4*. Colored nodes indicate significantly overrepresented GO terms (Benjamini-Hochberg-corrected $P < 0.05$). Scale bars indicate P-values.

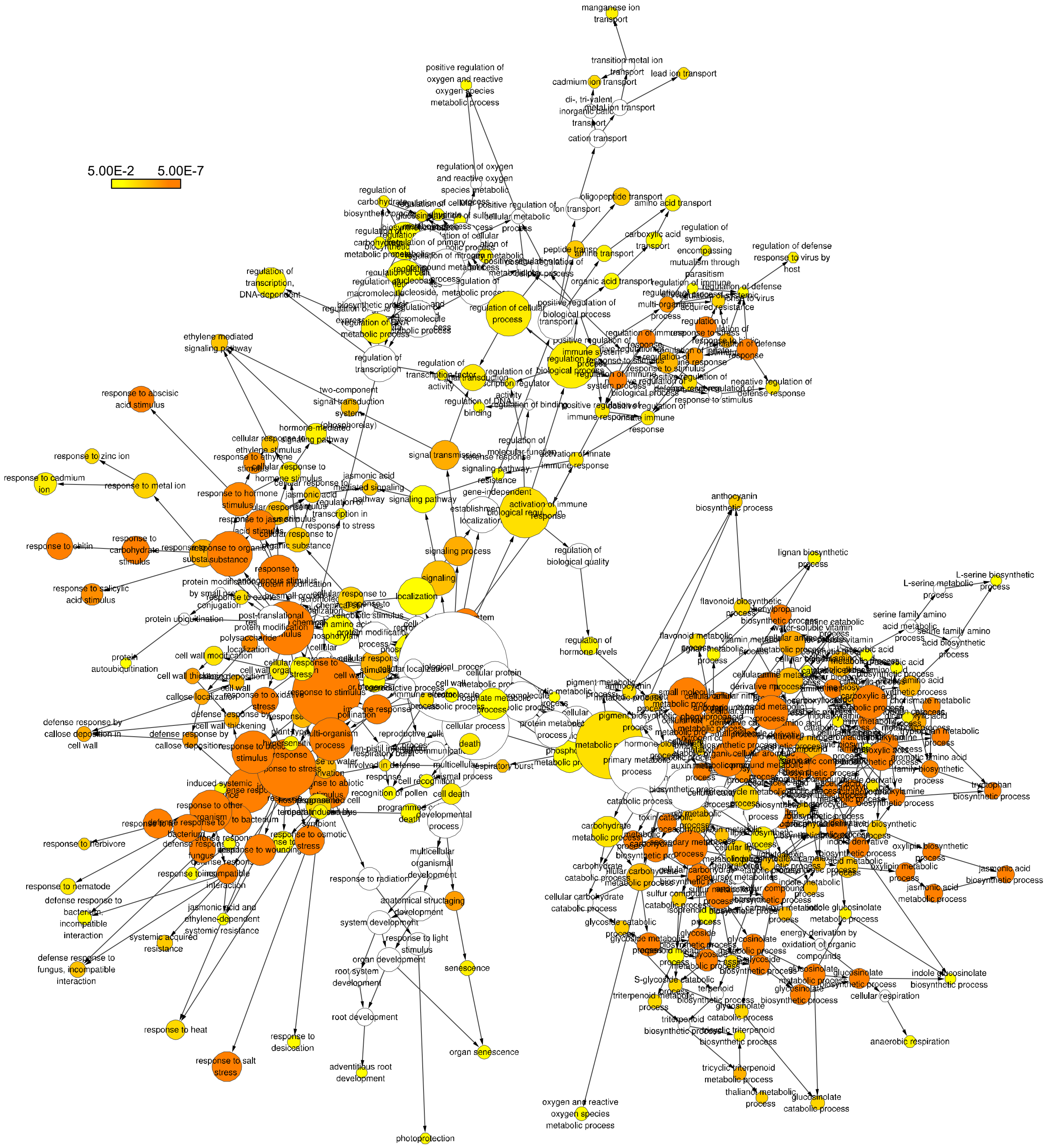

Supplementary Figure 9. Analysis of GO biological process using up-regulated genes in *fer-4* mutants.

RNA-sequencing was performed and differentially regulated genes were analyzed as described in **Supplementary Figure 6**. GO biological process analysis was performed using up-regulated genes in *fer-4*. Colored nodes indicate significantly overrepresented GO terms (Benjamini-Hochberg-corrected *P* < 0.05). Scale bars indicate *P*-values.

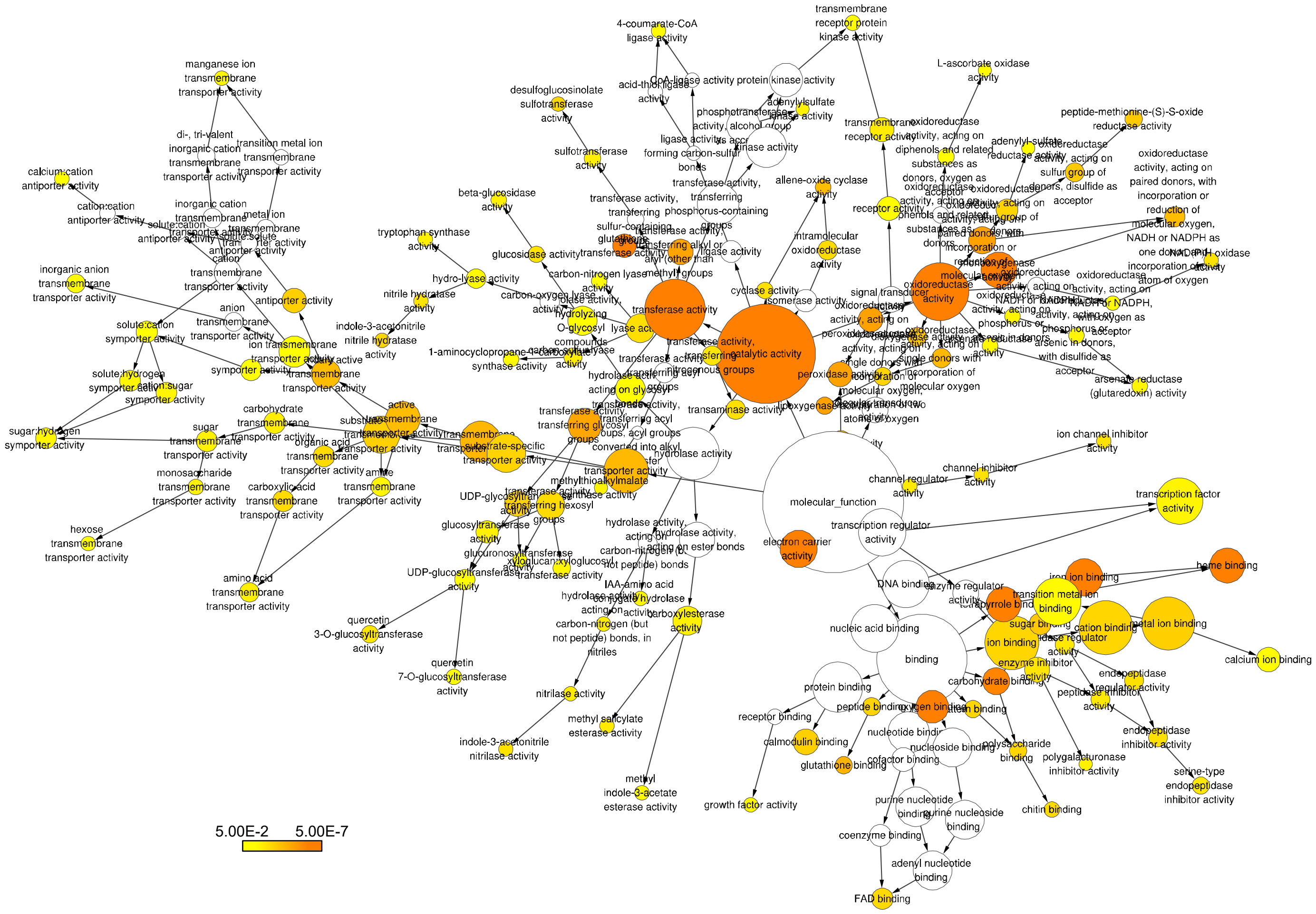

Supplementary Figure 10. Analysis of GO molecular function using up-regulated genes in *fer-4* mutants.

RNA-sequencing was performed and differentially regulated genes were analyzed as described in **Supplementary Figure 6**. GO molecular function analysis was performed using up-regulated genes in *fer-4*. Colored nodes indicate significantly overrepresented GO terms (Benjamini-Hochberg-corrected *P* < 0.05). Scale bars indicate *P*-values.

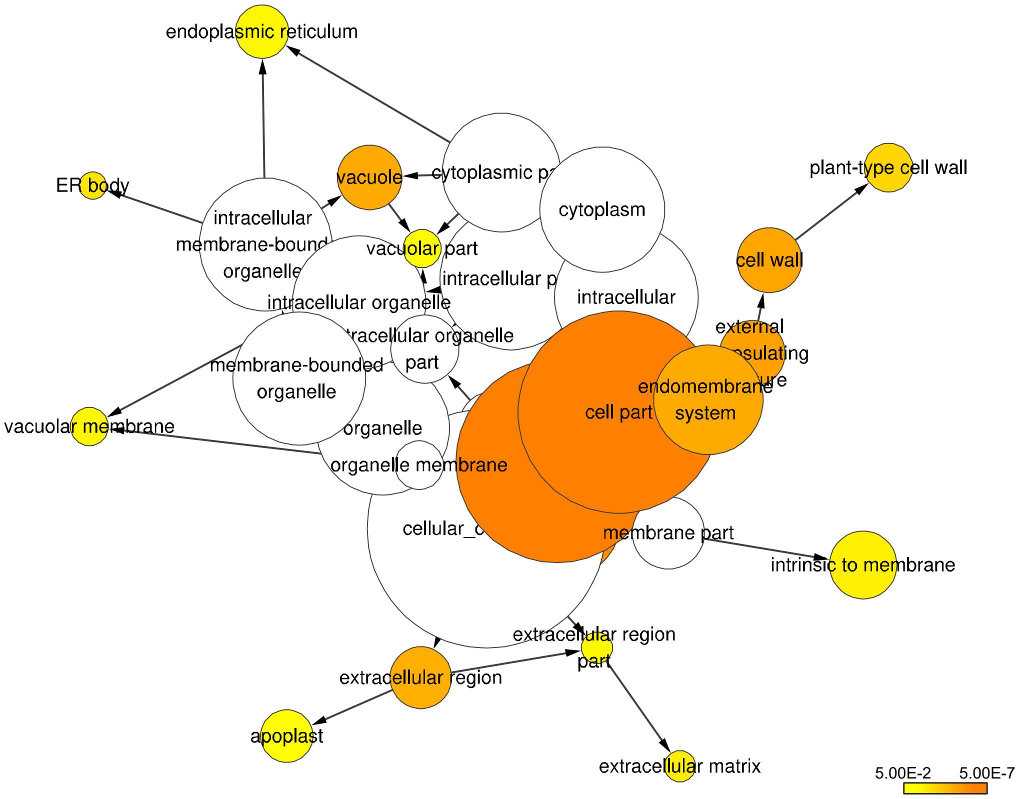

Supplementary Figure 11. Analysis of GO cellular component using up-regulated genes in *fer-4* mutants.

RNA-sequencing was performed and differentially regulated genes were analyzed as described in **Supplementary Figure 6**. GO cellular component analysis was performed using up-regulated genes in *fer-4*. Colored nodes indicate significantly overrepresented GO terms (Benjamini-Hochberg-corrected *P* < 0.05). Scale bars indicate *P*-values.

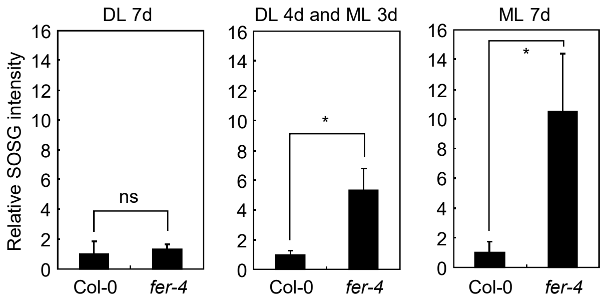

Supplementary Figure 12. Measurement of SOSG intensities in **FIGURE 5C**.
SOSG fluorescence intensities in **FIGURE 5C** were measured using ImageJ software. Intensity values in Col-0 seedlings were set to 1. Four to five biological replicates were averaged and statistically analyzed using Student's *t*-test (*, $P < 0.05$; difference from Col-0). Whiskers indicate SD.

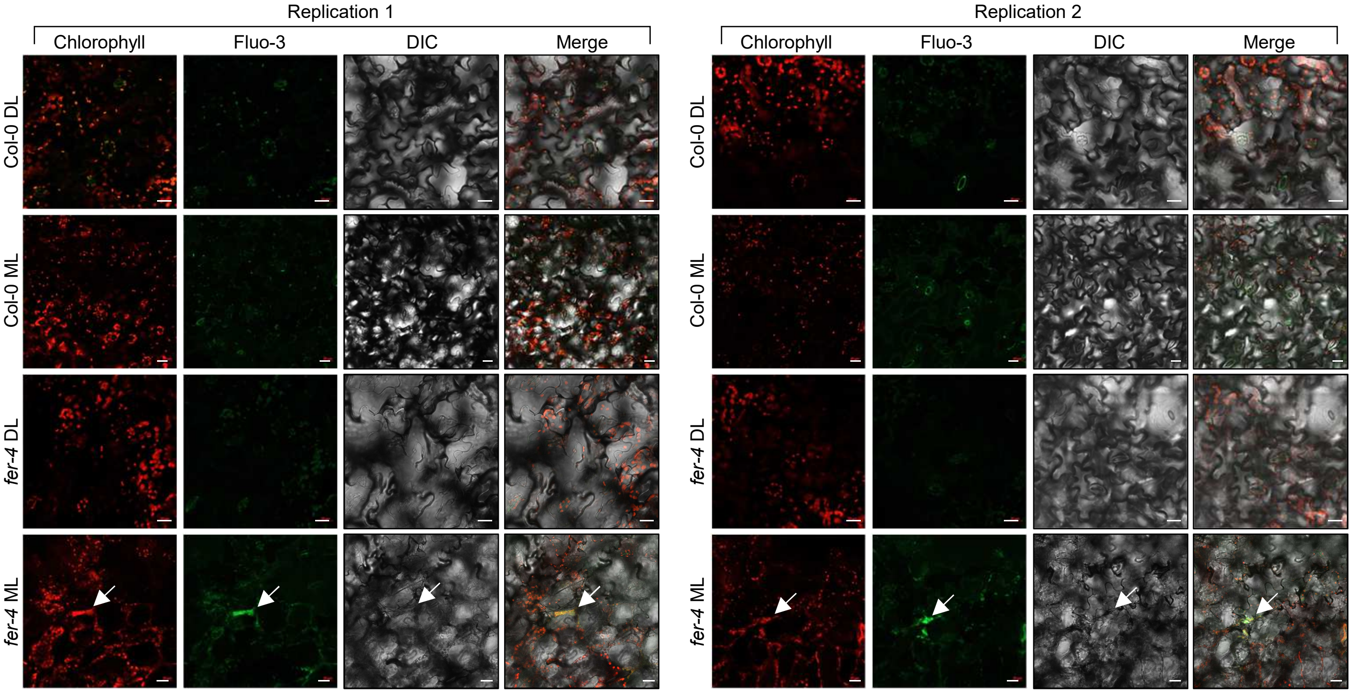

Supplementary Figure 13. Ca^{2+} signals under different light intensities.
The Col-0 and *fer-4* seedlings were grown on MS-agar plates at DL or ML for six days under long-day conditions. Seedlings were then subjected to Fluo-3 AM staining. Abaxial side of the leaves was used for analyzing fluorescence signals. Confocal microscope was used. Arrows indicate regions that show strong Fluo-3 AM fluorescence signals. DIC, differential interference contrast. Size markers indicate 20 μm.

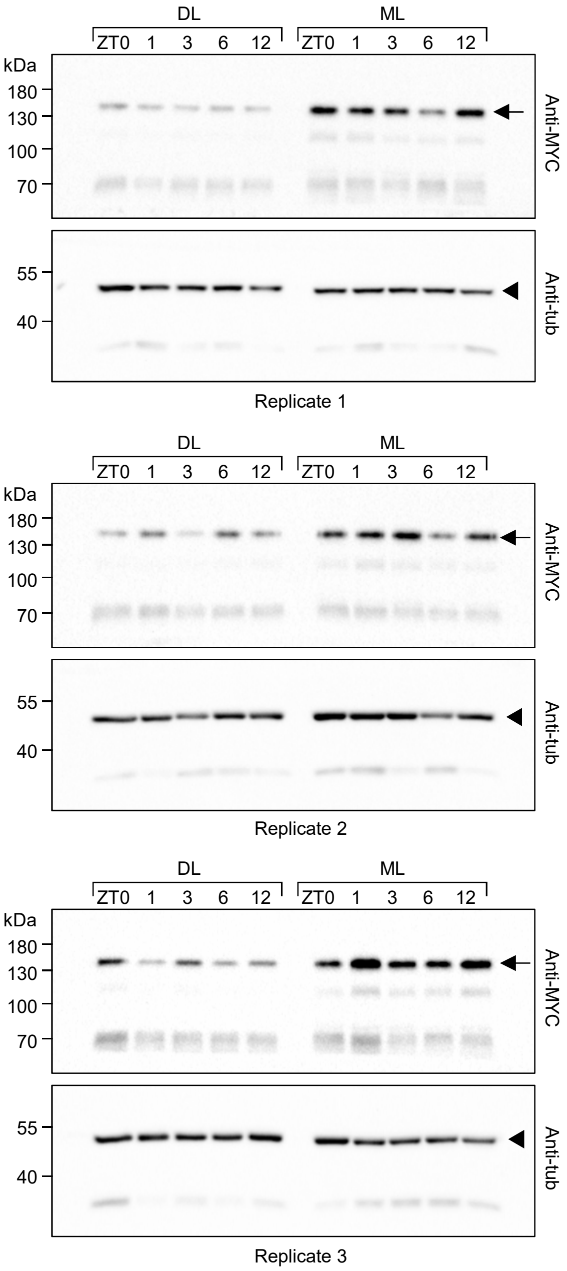

Supplementary Figure 14. Immunoblot images of biological replicates in **FIGURE 9C**.
Aerial parts of the *FER*$_{pro}$*:FER-MYC* transgenic seedlings were used for immunoblot assays. Arrows indicate FER-MYC fusion proteins and arrowheads indicate α-tubulin.

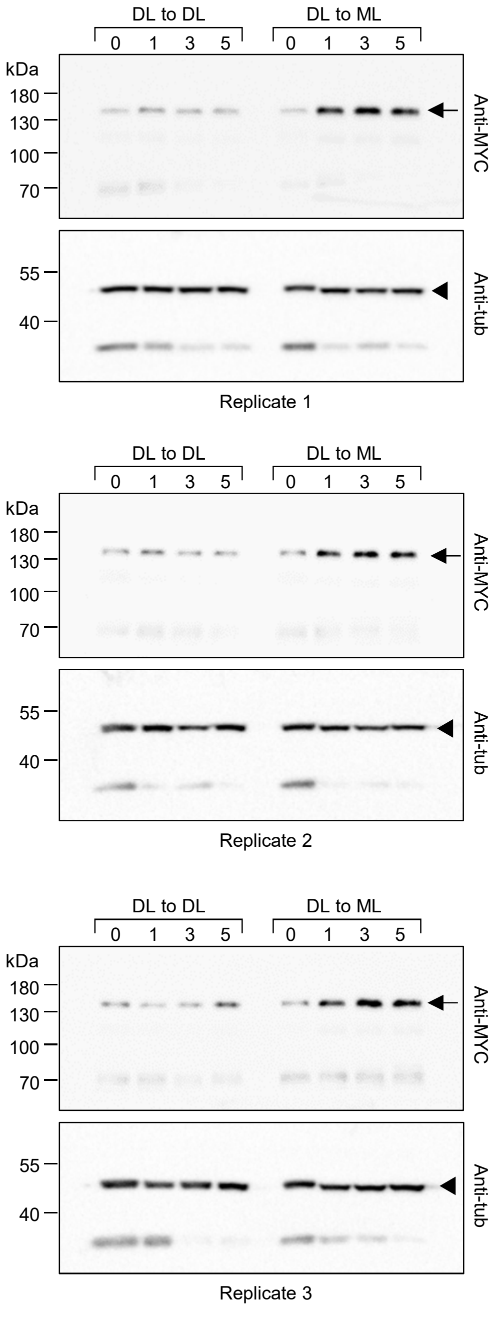

Supplementary Figure 15. Immunoblot images of biological replicates in **FIGURE 9E**.
Aerial parts of the *FER*$_{pro}$*:FER-MYC* transgenic seedlings were used for immunoblot assays. Arrows indicate FER-MYC fusion proteins and arrowheads indicate α-tubulin.

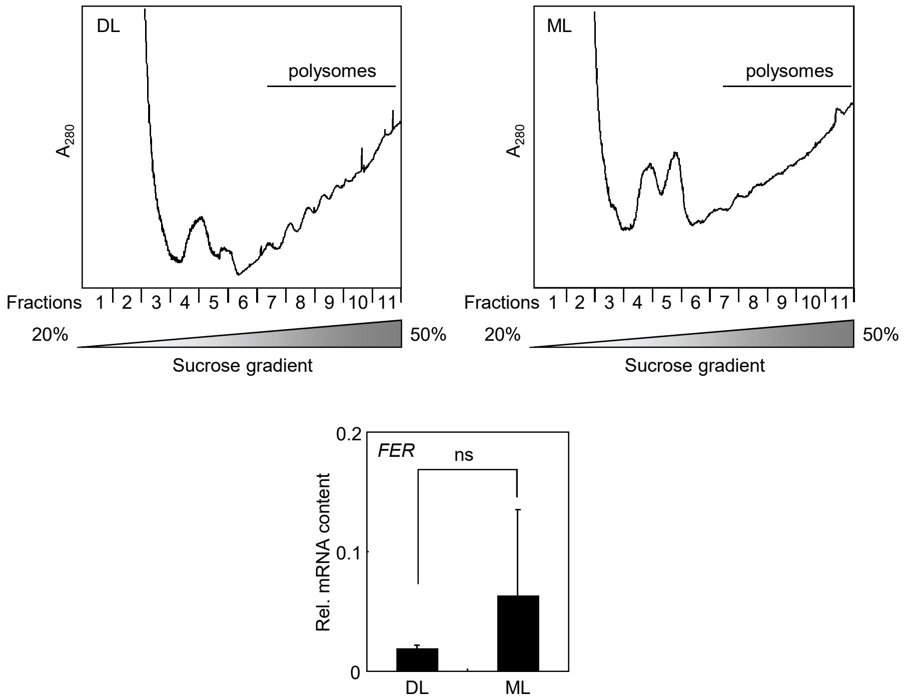

Supplementary Figure 16. Analysis of polysome-associated FER mRNAs.
Seven-day-old Col-0 seedlings grown on MS-agar plates under long-day conditions at DL or ML were used for polysome fractionation. Average ribosome profiles of three biological replicates were displayed and polysome fractions (8-11 fractions) were marked (upper panel). The relative proportion of the FER mRNAs associated with polysomes in the total mRNA was calculated (bottom panel). Three biological replicates were averaged and statistically analyzed using Student's *t*-test (*, $P < 0.05$). Whiskers indicate SD.

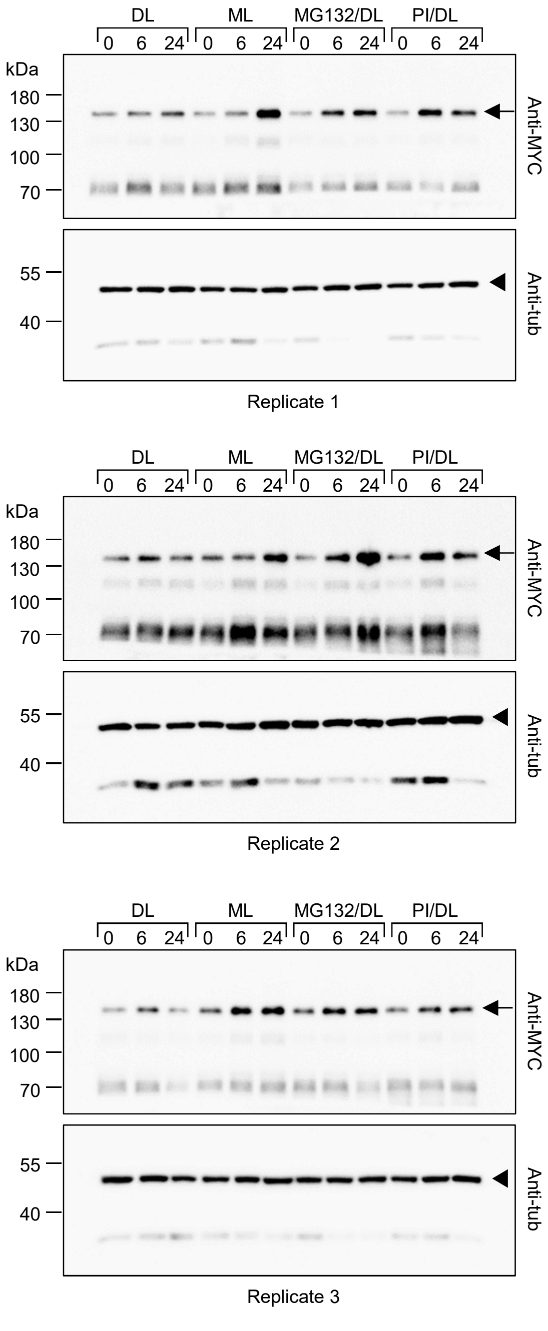

Supplementary Figure 17. Immunoblot images of biological replicates in **FIGURE 9G**.
Aerial parts of the *FER*$_{pro}$*:FER-MYC* transgenic seedlings were used for immunoblot assays. Arrows indicate FER-MYC fusion proteins and arrowheads indicate α-tubulin.

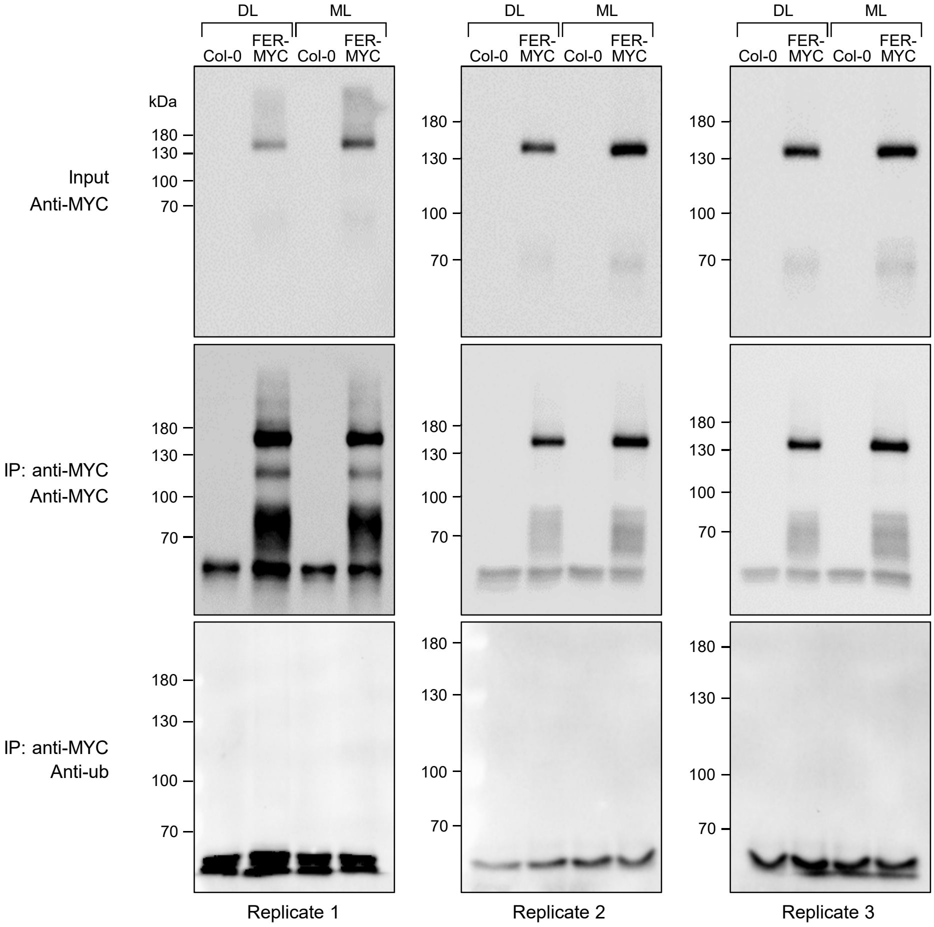

Supplementary Figure 18. Immunoblot images of biological replicates in **FIGURE 9H**.
The Col-0 and FER_{pro}:*FER-MYC* (FER-MYC) seedlings were used for immunoprecipitation and immunoblot assays.

Primers	Sequences	Usage
pFER AvrII-F	ataaatcctaggGAAAAGTTAAGAGTGGGAACTGGGAAGA	DNA cloning
pFER BamHI-R	atttatggatccCGATCAAGAGCACTTCTCCGGGAAGAT	″
FER BamHI-F	ataaatggatccATGAAGATCACAGAGGGACGATTCCG	″
FER AscI-R	atttatggcgcgccACGTCCCTTTGGATTCATGATCTGAGAA	″
APX2-F	TGTCTGGTGGACACACCTTG	RT-qPCR
APX2-R	AGAAGAGCCTTGTCGGTTGG	″
KIN1-F	CGGATTGGCAAAAGCAGGTC	″
KIN1-R	TCTAGTAGCACGACCCCGAA	″
RD29B-F	GTGGAGAAGGAATGGTGGGG	″
RD29B-R	CACTTCCACCGGAATCCGAA	″
ABI1-F	TCTAGAGCCGTTCTTTGCCG	″
ABI1-R	TCTAGAGCCGTTCTTTGCCG	″
ABI2-F	TGCAACGGTGAATCTAGGGT	″
ABI2-R	CGGAGATATCCACACCTGCC	″
ABI3-F	GACGTGGGTAACCTCGGAAG	″
ABI3-R	AACCTGTAGCGCATGTTCCA	″
ABI4-F	AAACGCAAAGGCAAAGGTGG	″
ABI4-R	GCGGTTGCGAAAGTACCAAG	″
ABI5-F	GGTGAAGGCTGGTGTGGTTA	″
ABI5-R	AGCTTGACCCGGGAATGAAG	″
FER-F	CCCAGTTAACAAGCCGGAGT	″
FER-R	AGTAGTCGGGCGTAGGACTT	″
eIF4A-F	TGACCACACAGTCTCTGCAA	″
eIF4A-R	ACCAGGGAGACTTGTTGGAC	″

Supplementary Table 2. Primers used in this work.
The primers used were designed using the Primer Blast (https://www.ncbi.nlm.nih.gov/tools/primer-blast). F, forward primer; R, reverse primer.
